# Histone Hypervariants H2A.Z.1 and H2A.Z.2 Play Independent and Context-Specific Roles in Neuronal Activity-Induced Transcription of *Arc/Arg3.1* and Other Immediate Early Genes

**DOI:** 10.1523/ENEURO.0040-17.2017

**Published:** 2017-08-24

**Authors:** Carissa J. Dunn, Pushpita Sarkar, Emma R. Bailey, Shannon Farris, Meilan Zhao, James M. Ward, Serena M. Dudek, Ramendra N. Saha

**Affiliations:** 1 Molecular and Cell Biology Unit, School of Natural Sciences, University of California at Merced, Merced, CA 95343; 2 Laboratory of Neurobiology, NIEHS, NIH, Research Triangle Park, NC 27709; 3 Integrative Bioinformatics Support Group, NIEHS, NIH, Research Triangle Park, NC 27709

**Keywords:** Arc, epigenetics, H2A.Z, H2A.Z.1, H2A.Z.2, histone

## Abstract

The histone variant H2A.Z is an essential and conserved regulator of eukaryotic gene transcription. However, the exact role of this histone in the transcriptional process remains perplexing. In vertebrates, H2A.Z has two hypervariants, H2A.Z.1 and H2A.Z.2, that have almost identical sequences except for three amino acid residues. Due to such similarity, functional specificity of these hypervariants in neurobiological processes, if any, remain largely unknown. In this study with dissociated rat cortical neurons, we asked if H2A.Z hypervariants have distinct functions in regulating basal and activity-induced gene transcription. Hypervariant-specific RNAi and microarray analyses revealed that H2A.Z.1 and H2A.Z.2 regulate basal expression of largely nonoverlapping gene sets, including genes that code for several synaptic proteins. In response to neuronal activity, rapid transcription of our model gene *Arc* is impaired by depletion of H2A.Z.2, but not H2A.Z.1. This impairment is partially rescued by codepletion of the H2A.Z chaperone, ANP32E. In contrast, under a different context (after 48 h of tetrodotoxin, TTX), rapid transcription of *Arc* is impaired by depletion of either hypervariant. Such context-dependent roles of H2A.Z hypervariants, as revealed by our multiplexed gene expression assays, are also evident with several other immediate early genes, where regulatory roles of these hypervariants vary from gene to gene under different conditions. Together, our data suggest that H2A.Z hypervariants have context-specific roles that complement each other to mediate activity-induced neuronal gene transcription.

## Significance Statement

Epigenetic regulation of activity-induced gene transcription is pivotal in mediating neuronal responses that underlie several transcription-dependent downstream brain processes, yet it remains poorly understood. Thus, understanding roles of such epigenetic processes and their core components, such as variant histone H2A.Z, is necessary to comprehend brain development and function. In vertebrates, H2A.Z has two hypervariants, H2A.Z.1 and H2A.Z.2, which are encoded by different genes and differ by only three amino acids. Despite such similar sequences, we provide evidence suggesting that they regulate nonoverlapping gene cohorts in neurons and play context-specific, nonredundant roles in activity-induced transcription of immediate early genes. Together, our findings represent a substantial departure from the current H2A.Z biology by shifting the focus to independent contributions of H2A.Z hypervariants.

## Introduction

Activity-induced gene transcription, an integral component of neural plasticity, is conducted at the chromatin level by enabling the transcription machinery to access and “read-out” DNA (Greer and Greenberg, 2008). Several activity-induced neuronal immediate early genes (IEGs) are accessed independent of signal by a transcriptionally engaged RNA polymerase II (Pol II) that pauses proximal to the promoter until an activity signal is received (Saha et al., 2011; Adelman and Lis, 2012). Activity-induced “turn-on” signals release the paused Pol II, which then efficiently transcribes the gene by running through spools of nucleosomes (productive elongation). Nucleosomes, the fundamental chromatin unit, regulate Pol II elongation by acting as either barriers or facilitators (Owen-Hughes and Gkikopoulos, 2012; Teves et al., 2014). Such activity-dependent nucleosomal role selection primarily depends on post-translational modifications of the histone tails and nucleosomal remodeling, including turnover of different histone variants (Michod et al., 2012).

In mammals, three of the four canonical histones, H2A, H2B, and H3, have diversified into several variant histones with distinct amino acid sequences that can influence transcriptionally relevant nucleosomal properties and dynamics (Weber and Henikoff, 2014). Recently, a few studies have unveiled important roles of histone variants H2A.Z and H3.3 in neuronal gene transcription underlying brain development and function. For example, H2A.Z, which is conserved from yeast to human and has been implicated in both transcriptional activation and repression (Soboleva et al., 2014), plays a role in memory consolidation and cerebellar development in the brain (Zovkic et al., 2014; Yang et al., 2016). Similarly, H3.3, which differs from canonical H3 histones by only four to five amino acids (Filipescu et al., 2013), regulates embryonic and adult neuronal gene expression patterns, thereby playing an essential role in plasticity and cognition (Maze et al., 2015). This example of H3.3 illustrates how very small dissimilarities in sequences of two closely related histone proteins may produce large differences in neuronal gene transcription (Maze et al., 2014).

**Figure 1.**
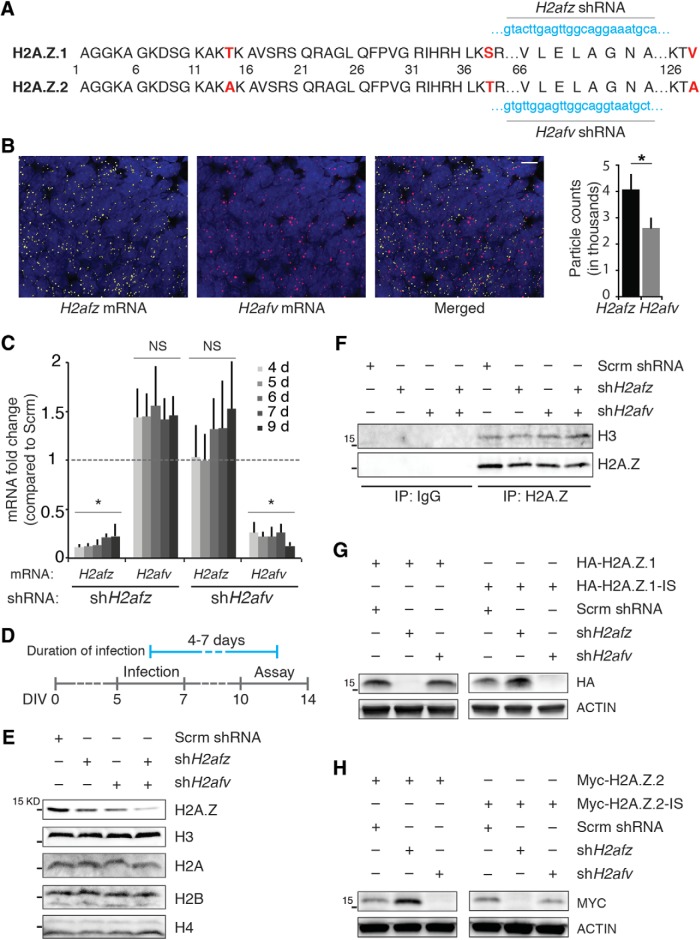
Validation of H2A.Z hypervariant-specific RNAi. ***A***, H2A.Z.1 and H2A.Z.2 amino acid sequences are depicted where three different amino acid residues are indicated in red. The target sequences of sh*H2afz* and sh*H2afv* are noted in blue. ***B***, left, Representative images of smFISH of *H2afv* and *H2afz* in E18 rat cortex. Right, Quantification of *H2afz* and *H2afv* molecules; particle count per 212 μm^2^. *N* = 3, *p < 0.05 (one-tailed unpaired *t* test). Scale bar, 10 μm. ***C***, Cortical neurons in culture were infected with lentiviruses delivering either sh*H2afz* or sh*H2afv* and total RNA was collected after indicated number of days. Compared to control neurons, changes in *H2afz* and *H2afv* mRNA levels were determined by qPCR, normalized to *Gapdh* mRNA levels (internal control) and are depicted here as fold change. NS, not significant. *p < 0.05. *N* = 3–6. ***D***, Timeline of infection and treatment for all assays performed in dissociated cortical neurons. Cells were infected with lentiviruses between 5 and 7 d after plating (gray line), and assays were performed between 4 and 7 d after infection (between DIV10 and DIV14; blue line). ***E***, Total acid-extracted histone from neurons infected with lentiviruses delivering either sh*H2afz* or sh*H2afv* or both for 6–7 d, resolved by electrophoresis, and blotted for indicated histone. *N* = 3. Note the cumulative effect of depleting both hypervariants when probed with total H2A.Z antibody. ***F***, IP of nucleosomal H2A.Z from nuclear extract of neurons infected with lentiviruses delivering either sh*H2afz* or sh*H2afv* or both for 6 d resolved by electrophoresis. *N* = 3. ***G***, ***H***, Neurons were coinfected with indicated constructs for 4–5 d and nuclear extracts were resolved by electrophoresis. IS, insensitive; shRNA target regions were swapped in these constructs. *N* = 3.

Similar small differences also exist within the H2A.Z histone class. In vertebrates, H2A.Z has two very closely related paralogs that have been referred to as “hypervariants”: H2A.Z.1 (formerly H2A.Z) and H2A.Z.2 (formerly H2A.V; Talbert et al., 2012). Two nonallelic genes, *H2afz* and *H2afv*, which are driven by independent promoters on different chromosomes, encode these H2A.Z hypervariants (Dryhurst et al., 2009; Matsuda et al., 2010). H2A.Z.1 and H2A.Z.2 differ by only three amino acids, which are located far apart on the polypeptide (Fig. 1*A*) and are structurally similar to their respective counterpart in that there are no usable difference in their antigenicity (e.g., hydrophobic vs hydrophilic, ring structure vs linear, or large vs small in residue size). Thus, there are currently no antibodies available to distinguish between H2A.Z.1 and H2A.Z.2. Due to this limitation, our understanding of H2A.Z is most likely a portmanteau of both hypervariant functions. Such a composite picture of their function would not be a concern if these hypervariants were functionally redundant and homogeneous (Soboleva et al., 2014). However, a few structural and functional features suggest the contrary. Recently resolved crystal structures of H2A.Z-containing nucleosomes revealed subtle differences in the H2A.Z.1 and H2A.Z.2 L1 loop (Horikoshi et al., 2013), but it remains unknown whether such differences manifest any functional consequences. In H2A.Z.1^−/−^ mice, which are embryonic lethal (Faast et al., 2001), H2A.Z.2 is apparently unable to compensate for the loss of its twin isoform, thereby suggesting a unique role for H2A.Z.1 during early embryonic development. Similar functional inequality of H2A.Z.1 and H2A.Z.2 has also been recently reported in melanoma cells (Vardabasso et al., 2015). Taken together, there remains an untested possibility that these two H2A.Z hypervariants may not be functionally identical, but instead have discrete nonoverlapping roles (functional specificity) in gene transcription. We tested this hypothesis in neurons and report here that H2A.Z.1 and H2A.Z.2 have independent and context-specific roles in basal and neuronal activity-induced gene transcription.

## Materials and Methods

### Plasmids and subcloning

Several web-based software programs were used to design eight (four each) hypervariant-specific shRNA for RNAi. The shRNA were inserted into pLKO.1-puro (designed by RNAi consortium or TRC; obtained from Addgene) following the protocol on the Addgene website. Self-inactivating HIV lentivirus particles were produced by transfecting 293T cells with the shRNA vector, envelope (pMD2.G; Addgene), and packaging plasmids (psPAX2; Addgene). For construction of exogenous tagged constructs, DNA sequences encoding HA-H2A.Z.1, HA.IS1, HA-ISB, MYC-H2A.Z.2, MYC-IS2, and MYC-ISB were generated each with 5′ EcoRI and 3′ NotI restriction sites, using Life Technologies GeneArt Gene Synthesis “strings.” These EcoRI-NotI DNA fragments were cloned into the System Biosciences lentiviral plasmid CD811A-1, using the corresponding restriction sites on the vector. Subsequently, DNA constructs were characterized by restriction mapping using EcoRI and NotI enzymes (New England Biolabs), and inserted elements were validated by sequencing. These constructs were packaged into lentiviruses as mentioned above.

### Dissociated neuronal culture, RNAi, and cell treatment

Cultures of cortical neurons were prepared from embryonic day 18 (E18) Sprague Dawley rats of either sex. All animal procedures were performed in accordance with the National Institute of Environmental Health Sciences (NIEHS) and the University of California Merced animal care committee’s regulations [NIEHS Institutional Animal Care and Use Committee (IACUC) approval: ASP#01-21; and University of California Merced IACUC approval: ASP#13-0007 and ASP#16-0004]. Dissociated cortical neurons were plated in Neurobasal medium (Invitrogen) supplemented with 25 μM glutamate (Sigma-Aldrich), 0.5 mM L-glutamine (Sigma-Aldrich), either B27 (Invitrogen) or NS21, and maintained in a similar medium without the glutamate. NS21 was prepared in the laboratory as previously described (Chen et al., 2008). Neurons were routinely used between 10 and 14 d *in vitro* (DIV), a stage when synapses have formed and are effective. For infection with recombinant lentiviruses, the viral supernatant was diluted in neuronal media and cells were infected between DIV5 and DIV7 at a multiplicity of infection ranging from 2 to 5 for 4–7 d. To induce gene transcription under resting conditions using synaptic circuits, we cotreated neurons with 50 μM bicuculline (Bic; Sigma-Aldrich) and 75 μM 4-aminopyridine (4AP; Acros Organics; Papadia et al., 2005). To induce gene transcription extrasynaptically, we blocked neuronal activity with 1–2 μM tetrodotoxin (TTX; Calbiochem) and activated the MAP kinase (MAPK) pathway (via PKC) with 1 μM phorbol ester myristate (PMA; Sigma-Aldrich; Schultz et al., 1997). To induce gene transcription at the intranuclear level, neurons were treated with 100 μM 5, 6-dichloro-1-β-D-ribofuranosyl-benzimidazole (DRB; Sigma-Aldrich) alone, or in combination with 250–500 nM triptolide (Trip; Tocris) for 1 h followed by washout with preconditioned media. To induce genes after homeostatic changes, a previously described protocol using prolonged treatment of TTX for 48 h followed by its washout was used (Saha et al., 2011).

### Electrophysiological recordings

Whole-cell patch-clamp recordings were performed on dissociated cortical neurons coexpressing GFP and shRNA by an experimenter blinded to the identity of the treatment. Miniature EPSCs (mEPSCs) were assessed for frequency and amplitude as detailed previously (Saha et al., 2011). For visualization of infected neurons using GFP coexpression, the shRNA component was subcloned from the pLKO.1 to the pLKO.3G backbone as detailed previously (Saha et al., 2011). To ensure adequate knockdown, PCR was performed in sister neuronal cultures with the same batch of viruses to confirm their efficacy.

### RNA extraction and gene transcription quantification

Total RNA was isolated from dissociated neurons using the RNeasy Mini kit (QIAGEN) with in-column DNase (QIAGEN) digestion. cDNA was synthesized using MuLV reverse transcriptase (Promega), random primers (Promega), oligo dTs (Promega), and RNase inhibitors (Thermo Scientific). qPCR was performed using iTaq Universal Sybr Green Supermix (Bio-Rad) and the Bio-Rad CFX Connect real-time PCR Detection System. Pre-mRNA was estimated as described previously (Saha, et al., 2011). Rat PCR primers used in this study are listed in Table 1.

**Table 1.** List of primers used in the study

Target	Forward primer	Reverse primer
*Arc* pre-mRNA	GAATTTGCTATGCCAACTCACGGG	AGTCATGGAGCCGAAGTCTGCTTT
*Btg2* pre-mRNA	CTCTCTCTCTTGTTTCCTCCACAG	TGTGGTTGATGCGGATACAGCGAT
*cFos* pre-mRNA	ACAGCCTTTCCTACTACCATTCCC	CTGCACAAAGCCAAACTCACCTGT
*Cyr61* pre-mRNA	ATGTATGAGTTTCAGCGTGTGGCG	GTCTGCCTTCTGACTGAGCTGTAA
*Dusp1* pre-mRNA	CTCTACGACCAGGTTAGTAGGAGT	ACAGCCGCTTTCTCTATTCTCCCT
*Dusp6* pre-mRNA	TCCTGTGCCTCTCACAAGCTGAAA	AACTTACTGAAGCCACCTGCCAGA
*Fbxo33* pre-mRNA	GCATCTACTTGGAGCTGGTGTTGT	TCCACGCAAGCCTACCTGTTGTT
*Gadd45g* pre-mRNA	ACTCACGGCGCTTGTTCTTTCACA	ATTCAGGACTTTGGCGGACTCGTA
*GAPDH* pre-mRNA	AACATGCACAGGGTACTTCGAGGA	ACGACATACTCAGCACCAGCATCA
*Npas4* pre-mRNA	GTTGCATCAACTCCAGAGCCAAGT	ACATTTGGGCTGGACCTACCTTCA
*Nr4a3* pre-mRNA	ATGGAGTGTCAACTGGCTTCTGAG	GCCATAAGTCTGCGTGGCATAAGT
*GAPDH* mRNA	AGAGACAGCCGCATCTTCTTG	GGTAACCAGGCGTCCGATAC
*H2Afz* mRNA	GAAGAAAGGACAACAGAAGACTGT	CAGCTGTTAAGAGTATTTAGAGTCC
*H2Afv* mRNA	ACCCTATGCTCCCGTGTGTTAGAA	AGGCAAAGATCAGCACCAACTCTG
18s rRNA	CATTCGAACGTCTGCCCTAT	GTTTCTCAGGCTCCCTCTCC
*ANP32e* mRNA	TCAGAAGTAGGAGAGGGAGAAG	CCTTGGAGGGTCTAATCATCATC

### Expression microarray and data analysis

Gene expression analysis was performed using RNA obtained from four biological replicates. The Agilent Whole Rat Genome 4x44 multiplex format oligo arrays (014879, Agilent Technologies) were used following the Agilent 1-color microarray-based gene expression analysis protocol. Using 500 ng of total RNA, Cy3-labeled cRNA was produced according to the manufacturer’s protocol. For each sample 1.65 μg of Cy3-labeled cRNAs were fragmented and hybridized for 17 h in a rotating hybridization oven. Slides were washed and then scanned with an Agilent Scanner. Data were obtained using the Agilent Feature Extraction software (v9.5), using the 1-color defaults for all parameters. The Agilent Feature Extraction Software performed error modeling, adjusting for additive and multiplicative noise. Then, “gProcessedSignal” values were extracted for each probe and sample and used as the baseline expression measurements and quantile normalization was performed to normalize the samples. Probe-based expression data were collapsed down to a single measurement per gene by taking the median of all probes associated with each gene. Next, the average expression was calculated across all samples for each gene and the bottom 10% of genes with the lowest average expression was removed. Differentially expressed genes (DEGs) were identified by first performing a two-sample Student’s *t* test for each gene followed by Benjamini Hochberg correction. Genes were defined as differentially expressed if they had *p* < 0.001 and a fold change of 1.25 or greater. For the heat map depicting expression of these DEGs, the expression values were normalized to have a mean of zero and a standard deviation of one, and hierarchical clustering of these genes was performed. GO terms that were enriched for these DEGs were identified using the online tool DAVID (http://david.ncifcrf.gov/). GEO accession number for these datasets is GSE96886.

### Sample preparation for electrophoresis

Neurons were lysed in ice-cold 1× RIPA buffer (25 mM Tris, pH 7.5, 150 mM NaCl, 1% Na-deoxycholate, 0.1% SDS, and 1% NP-40) and supplemented with 1:100 protease inhibitor cocktail (Sigma-Aldrich). Lysed neurons were sheared by sonication, cell debris pelleted at 15000 rpm for 5 min at 4°C, and the clarified supernatant transferred to a prechilled 1.7 ml microcentrifuge tube. Total cell extracts were denatured at 95°C for 5 min, using either home-made 5× Laemmli buffer, BOLT 4× Sample buffer (Life Technologies) and BOLT 10× reducing agent (Life Technologies), 2×-, or 4× Laemmli sample buffer (both from Bio-Rad). To obtain synaptic fractions, cell pellets were treated with 1 ml ice-cold Syn-PER reagent (Thermo Scientific catalogue number 87793) and supplemented with 1:100 protease inhibitor cocktail (Sigma-Aldrich). Neurons were incubated on ice for 10 min, gently triturated 10 times, centrifuged at 1200 × *g* for 10 min at 4°C, and supernatant was transferred to ice-cold microcentrifuge tubes, then recentrifuged at 15,000 × *g* for 20 min at 4°C. The pellet was then resuspended in 20 µl 2× Laemmli sample buffer (Bio-Rad). Acid-extracted histone fractions were prepared using a protocol described previously (Shechter et al., 2007). Histone pellets were denatured using 2× Laemmli sample buffer (Bio-Rad).

### Immunoprecipitations (IPs)

All IPs were performed overnight, at 4°C in 1× IP buffer (0.5% Triton X-100, 0.002 M EDTA, 0.02 M Tris pH 7.75, 0.15 M NaCl, and 10% glycerol), or in 1× IP buffer from the SimpleChIP Enzymatic Chromatin IP kit (Cell Signaling Technology catalogue number 9003). Proteins were isolated from overnight IPs by incubating with Pierce Protein A/G Magnetic Beads (catalogue number 88803) for 1 h at 4°C. Subsequently, the beads were washed three times with IP buffer, denatured, separated by electrophoresis, and immune blotted as noted below.

### Western blotting and imaging

Denatured protein samples were resolved on 4–20% (Bio-Rad catalogue number 4568095), 4–15% (Bio-Rad catalogue number 456–1083), 8–16% (Bio-Rad catalogue number 456–1103) Mini PROTEAN gels, or on BOLT 4–12% Bis-Tris Plus Gels (Life Technologies catalogue number NW04122BOX), in Tris/Glycine/SDS (Bio-Rad catalogue number 1610772), or 1× MOPS buffer (Life Technologies catalogue number B0001), respectively. Resolved proteins were transferred onto LF PVDF membrane, using the Bio-Rad TBT RTA kit and protocol (catalogue number 1704272). PVDF membranes were incubated at 4°C overnight with appropriate primary antibodies in 1× TBS-T with 0.5% BSA. The next day, membranes were washed three times in 1× TBS-T, probed with appropriate Alexa Fluor secondary antibodies (Life Technologies) for 30–45 min at room temperature, washed three times with 1× TBS-T, and imaged using Bio-Rad ChemiDoc Imaging System.

### Chromatin digestion

Micrococcal nuclease (MNase) digests of rat dissociated neurons were performed using the SimpleChIP Enzymatic Chromatin IP kit (Cell Signaling Technology catalogue number 9003). Cells were fixed using Thermo Scientific Pierce methanol-free formaldehyde (catalogue number PI28906). The manufacturer’s protocol was used with the exception that Thermo Scientific Pierce Micrococcal Nuclease (catalogue number PI88216) was used in place of CST MNase. Postdigest, samples were briefly sonicated at low settings to rupture the nucleus and then were either subjected to chromatin IP (ChIP), DNA extraction, or protein co-IP following the CST kit protocol.

### ChIP

Neurons were fixed with formaldehyde for 5–10 min, lysed in the sonication buffer (20 mM Tris, pH 7.8, 2 mM EDTA, 0.5 mM EGTA, 0.5% SDS, and inhibitors for proteases and phosphatases), and then sonicated for sixteen cycles (bursts and intervals of 30 s each) with the Bioruptor (Diagenode) to obtain 200- to 1000-bp genomic DNA fragments. A fraction of the sonicated sample was then immunoprecipitated overnight at 4°C in the 1× IP buffer with 2- to 5-μg antibody. Antigen-antibody complexes were immunoprecipitated with Pierce Protein A/G Magnetic Beads, washed once with low salt buffer, three times with high salt buffer, once with LiCl buffer, and once with Tris-EDTA buffer. Samples were reverse cross-linked at 65°C overnight, and chromatin DNA was eluted using the QIAquick Nucleotide Removal kit (QIAGEN). Eluted chromatin was quantified by qPCR.

### Immunocytochemistry and microscopy

Infected neurons were washed twice with 1× ice-cold PBS (Fisher Scientific). The cells were then incubated with 4% paraformaldehyde (Sigma-Aldrich) in 1× PBS for 15 min at room temperature and then washed twice with 1× PBS, permeabilized at room temperature for 20 min with 0.5% Triton X-100 (Fisher Scientific), washed twice with 1× PBS, and blocked for 30 min with 10% goat serum (Gibco) in 1× PBS. Cells were incubated at 4°C overnight in 3% goat serum in 1× PBS with primary antibodies at 1:500 dilution. The next day, the primary antibody solution was removed, and cells were washed three times with 0.05% Tween (Fisher) in 1× PBS (0.05% PBS-T), and incubated with appropriate secondary antibody in 3% goat serum in 1× PBS for 45 min, washed three times with 0.05% PBS-T, and cured overnight using ProLong Anti-Fade Gold with DAPI (Life Technologies). Images were captured with a Keyence BZ9000-E microscope at 20× magnification.

### Antibodies

Sheep anti-H2A.Z (Millipore, 09-862; RRID:AB_1587118), mouse anti-MYC (ThermoFisher Scientific, R95025; RRID:AB_2556560), rabbit anti-H3 (Cell Signaling Technology, 2650S; RRID:AB_2115124), mouse anti-β-ACTIN (ThermoFisher Scientific, AM4302; RRID:AB_2536382), rabbit anti-ARC (Synaptic Systems, 156003; RRID:AB_887694), rabbit anti-p-p44/42 MAPK (T202/Y204; Cell Signaling Technology, 4370S; RRID:AB_2315112), mouse anti-p44/42 MAPK (Erk1/2; L34F12; Cell Signaling Technology, 4696S; RRID:AB_390780), rabbit anti-HA (Cell Signaling Technology, 3724S; RRID:AB_1549585), mouse anti-MYC (Cell Signaling Technology, 2276S; RRID:AB_331783), rabbit anti-H2A (Cell Signaling Technology, 12349S; RRID:AB_2687875), mouse anti-H2B (Cell Signaling Technology, 2934S; RRID:AB_2295301), mouse anti-H4 (Cell Signaling Technology, 2935S; RRID:AB_1147658), rabbit anti-H2A.Z (Cell Signaling Technology, 2718S; RRID:AB_10694716), mouse anti-PSD95 (Antibodies Incorporated, 75-028; RRID:AB_2292909), rabbit anti-SH3 and multiple ankyrin repeat domains 3 (SHANK3) (Santa Cruz Biotechnology, SC30193; RRID:AB_2301759), goat anti-SHANK3 N16 (Santa Cruz Biotechnology, SC23547; RRID:AB_2187727), mouse anti-GluR2 (anti-GluA2; Millipore, MAB397; RRID:AB_2113875), mouse anti-HOMER1 (Synaptic Systems, 160011; RRID:AB_2120992), mouse anti-Synapsin1 (Synaptic Systems, 106001; RRID:AB_887805), rabbit anti-RPB1 (Cell Signaling Technology, 14958S; RRID:AB_2687876), rabbit anti-acetyl-H2A.Z Lys 5,7,11 (Millipore, ABE1363; RRID:AB_2687877), and rabbit anti-pSer5-RPB1 (Active Motif, 61085; RRID:AB_2687451).

### Single molecule fluorescent *in situ* hybridization (smFISH)

Rat brains were harvested at E18 and individually embedded in 22 × 22 mm cryomolds using optimal cutting temperature compound (OCT) and flash frozen in 2-methylbutane, which was cooled to −20°C on a dry ice/ethanol slurry. Brains were sectioned on a Leica cryostat at 20 μm and processed for single-molecule FISH according to the RNAscope Fluorescent Multiplex kit instructions (Advanced Cell Diagnostics). The following probes were used with the RNAscope fluorescent multiplex reagent kit: Rn-H2afv (20 ZZ probes targeting 414–1815 of NM_001106019.1; part# 495451), Rn-Hz1fz-C2 (13 ZZ probes targeting 12–792 of NM_022674.1; part# 495441-C2), 3-plex Positive Control Probe, rat (part# 320891), and 3-plex Negative Control Probe (part# 320871). Images were acquired on a Zeiss 880 confocal microscope using a 40× oil-immersion lens. The image acquisition parameters were set using 3-plex negative controls (cDNA probes against bacterial RNAs not present in rat tissue) in each of the three channels (fluorescein isothiocyanate, CY3 and CY5) so that any signal above the level of background was acquired. At least three 212 × 212 μm *z*-stack images (1-μm steps) of somatosensory cortex were acquired per rat. Images were processed and analyzed using FIJI software (NIH v.2). Maximum intensity projection images were thresholded to an average value per channel and particles were counted using the analyze particle function in FIJI. Background particle counts were subtracted from each value before taking the average particle count per channel per rat (*N* = 3).

### Nanostring assay and data analysis

NanoString probes were designed for indicated pre-mRNAs by NanoString technologies and assays were performed following the manufacturer’s protocol. Data were processed in R (version 3.3.2), using the limma package (version 3.30.8) from Bioconductor (version 3.4). Data were log2 transformed, then batch-adjusted within each experiment and time point to account for sample processing errors. The pre-mRNA measurements were normalized using pre-mRNA housekeeper genes. Sample quality and processing were reviewed and confirmed using MA-plots and heat maps at each step. Pairwise comparisons were performed using the limma-voom methodology as previously described. Statistical hits were defined by requiring one sample group mean log2 signal at least 2, absolute fold change above 1.5, and Benjamini Hochberg adjusted *p* < 0.01. Heat maps were generated using group mean log2 normalized abundances, centered to the indicated control group mean. Violin plots were generated using the same values, centered to the treated scrambled for the respective group of treatments. Gene class changes per experiment and time point were tested using group mean values in a nonparametric, paired Wilcoxon test, with gene as the pairing factor, to test for coordinated gene changes between treated shH2A.Z.1 or treated shH2A.Z.2 and the corresponding treated scrambled of the same experiment time point.

### Experimental replicates and statistics

Biological and technical replicates for each figure are indicated as *N* and *n*, respectively, in corresponding figure legends. Error bars represent standard error of mean throughout this article. Statistical comparison of datasets was performed with the two-tailed Student’s *t* test (with Bonferroni corrections for multiple comparisons), two-way ANOVA, or Wilcoxon paired nonparametric test, unless otherwise stated in the figure legend.

## Results

### H2A.Z hypervariants and strategy to target them independently

First, we asked if both H2A.Z isoforms, H2A.Z.1 and H2A.Z.2 (Fig. 1*A*), are expressed in the brain. Using the smFISH approach and specific probes that bind to *H2afz* or *H2afv*, we detected both H2A.Z isoforms in the E18 rat brain (Fig. 1*B*). While there were significantly more *H2afz* particles compared to *H2afv*, we observed that the majority of DAPI-positive cells expressed both RNAs. Further studies were performed in dissociated cells obtained from E18 rat brains.

Next, we used RNAi to target these hypervariants independently. Both genes have been duplicated during evolution and have multiple mammalian “gene copies” that have variable noncoding regions despite having the same coding region. For example, in rat, when compared to the annotated *H2afz* on chromosome 2, an unnamed “pseudogene” on chromosome 16 (rat rn6 location: 71438461-71439260) codes for an mRNA that retains the *H2afz* coding sequence in totality amid dissimilar noncoding regions. Therefore, to ensure knockdown of these probable pseudogene mRNAs as well, the coding region was targeted for RNAi. Initially, we designed four shRNA against each of the *H2afz* and *H2afv* mRNA coding regions and evaluated their knockdown efficacy by qPCR. Only one of these four shRNA pairs efficiently knocked down each hypervariant independently; others knocked down both hypervariants to some degree. Interestingly, these efficient shRNAs against *H2afz* (sh*H2afz*) and *H2afv* (sh*H2afv*) targeted the same region on the respective mRNA (Fig. 1*A*). These 24-bp target regions on *H2afz* and *H2afv* mRNAs differ by only 5 bp. Because of such scant difference, we tested whether sh*H2afz* and sh*H2afv* were indeed specific for their respective hypervariant targets.

Lentivirus-mediated delivery of sh*H2afz* to dissociated rat cortical neurons significantly knocked down *H2afz*, but not *H2afv*, mRNA at all tested times after viral infection (Fig. 1*C*). Similarly, *H2afv*, but not *H2afz*, mRNA was knocked down by sh*H2afv* (Fig. 1*C*). We also noted that loss of one hypervariant was not compensated by significantly enhanced expression of the other hypervariant. Because efficient knockdown was noted between days 4 and 7 after infection, we decided to use this timeline for the rest of our assays (Fig. 1*D*). At the protein level, as detected by a commercial anti-H2A.Z antibody that does not discriminate between hypervariants, infection with sh*H2afz* or sh*H2afv* viruses partially depleted endogenous H2A.Z levels without affecting the expression of other histones (Fig. 1*E*). Although we were unable to completely abolish H2A.Z signal on infection with both sh*H2afz* and sh*H2afv* viruses, H2A.Z protein depletion was more pronounced on coinfection. We suspect that the protein remaining after coinfection is nucleosomal H2A.Z protected in the heterochromatin (Fig. 1*E*,*F*). The cumulative effect of such coinfection suggests that neurons express both hypervariants, and that they are sensitive to their respective shRNA only.

To test the above stated possibility further, we coexpressed sh*H2afz* or sh*H2afv* with either tagged HA-H2A.Z.1 or tagged Myc-H2A.Z.2. Protein expression of HA-H2A.Z.1 was attenuated by sh*H2afz*, but not sh*H2afv*. Similarly, MYC-H2A.Z.2 protein expression was sensitive to sh*H2afv*, but not sh*H2afz* (Fig. 1*G*,*H*, left panels). To test sh*H2afz* and sh*H2afv* specificity even further, we exchanged their 24-bp target sequence in our exogenous constructs with the expectation that shRNA sensitivity of these constructs will also be reversed. As expected, HA-H2A.Z.1-IS, the *H2afz* mRNA with the sh*H2afv* 24-bp target sequence, is sensitive to sh*H2afv*, but not sh*H2afz*. Similarly, Myc-H2A.Z.2-IS is sensitive to sh*H2afz*, but not *shH2afv* (Fig. 1*G*,*H*, right panels). Taken together, we show that sh*H2afz* and sh*H2afv* are specific for *H2afz* and *H2afv* mRNAs respectively, and that we can efficiently knockdown each targeted hypervariant independently.

### H2A.Z hypervariant depletion alters expression of nonoverlapping sets of genes

**Figure 2.**
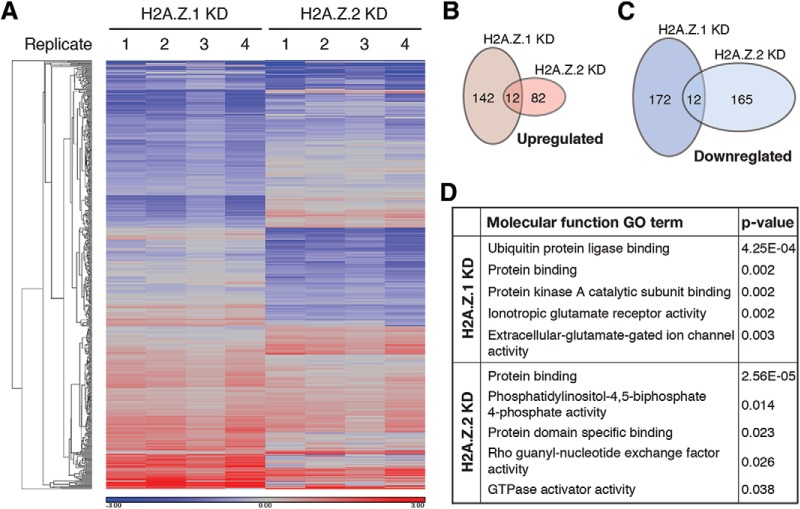
H2A.Z hypervariant depletion alters baseline expression of mostly nonidentical gene cohorts. ***A***, Heat map showing changes in gene expression as detected by microarray analysis after depletion of H2A.Z.1 and H2A.Z.2 compared to control neurons. *N* = 4. Genes showing >1.25-fold change with *p* < 0.001 are shown here. ***B***, ***C***, Venn diagrams depicting total number of genes that are either upregulated (***B***) or downregulated (***C***) after depletion of H2A.Z.1 or H2A.Z.2. Note the nonoverlapping nature of such gene sets. ***D***, Top five GO terms derived from molecular function gene ontology analysis (DAVID) for H2A.Z.1 and H2A.Z.2 target genes.

To examine the impact of individual H2A.Z hypervariant knockdown on basal level of neuronal gene transcription, we performed a genome-wide microarray analysis of RNA obtained from dissociated cortical neurons depleted of H2A.Z.1 or H2A.Z.2. Gene expression levels in these samples were compared to samples from neurons treated with a scrambled shRNA (referred to as control neurons hereafter). The degree of change in the mRNA levels for every gene, whose transcription was altered due to H2A.Z.1 or H2A.Z.2 deficiency (>1.25-fold), is plotted in Figure 2 as a heat map. A total of 338 genes were differentially expressed due to depletion of H2A.Z.1 (154 genes were upregulated and 184 were downregulated), while H2A.Z.2 depletion altered expression of 271 genes (94 genes were upregulated and 177 were downregulated). Only 24 genes were sensitive to depletion of either H2A.Z isoform (Fig. 2*B*,*C*; Tables 2–5). Such relative dearth of common genes affected by depletion of either hypervariant suggests that H2A.Z.1 and H2A.Z.2 regulate, directly or indirectly, independent gene cohorts. Functional gene ontology analysis of H2A.Z.1 and H2A.Z.2 target genes revealed that targets of the former are enriched for ionotropic postsynaptic receptors (Fig. 2*D*). Three of the top 10 molecular GO pathways belonged to this category. This finding contrasts with H2A.Z.2 target genes, where none of the hits were related to synaptic function. Interestingly, knockdown of H2A.Z.1, but not H2A.Z.2, led to altered expression of several candidate genes linked to psychiatric disorders such as autism and schizophrenia (*Neurexin1*, *Shank3*, *CaMK2G*, *Vamp7*, *Gsk3b*, *Tuba8*, *Grm8*, etc.).

**Table 2.** List of DEGs after H2A.Z.1 and H2A.Z.2 KD-part A (upregulated after H2A.Z.1 KD)

Symbol	Scrambled expression	shH2AZ.1 expression	log2 fold change	*p* value	FDR
Ace	4.140032802	5.231924578	1.091891777	2.67E—06	0.049404732
BF565662	3.628967203	5.376799174	1.747831971	1.69E—05	0.065026891
TC524990	8.870590012	10.29163907	1.421049062	1.88E—05	0.065026891
Ccdc23	14.47197211	15.09673068	0.624758569	2.34E—05	0.065026891
RGD1307943_predicted	5.328316737	7.637953753	2.309637017	3.41E—05	0.076745729
RGD1563203_predicted	10.11295573	10.66114527	0.548189542	3.52E—05	0.076745729
AW918709	11.15514358	12.49064688	1.335503299	4.75E—05	0.07711271
Ddx39	11.53226567	12.3812449	0.848979229	5.93E—05	0.07711271
CX570116	8.384790847	8.916443761	0.531652913	6.90E—05	0.07711271
BI293610	9.493734401	9.936922735	0.443188333	7.00E—05	0.07711271
ENSRNOT00000013636	10.29622089	11.25318643	0.956965537	7.45E—05	0.07711271
Slc13a5	8.198920861	9.995278642	1.796357781	7.61E—05	0.07711271
ENSRNOT00000028053	6.936499815	7.576197512	0.639697697	7.67E—05	0.07711271
Ddc	6.457510865	8.216773518	1.759262653	7.75E—05	0.07711271
Hdmcp	6.071940673	7.657559709	1.585619036	8.10E—05	0.07711271
CA339586	11.74191409	12.75911189	1.01719779	9.56E—05	0.07711271
Prlr	3.864580889	4.720040256	0.855459367	9.71E—05	0.07711271
TC533299	10.13831539	10.92955405	0.791238655	9.94E—05	0.07711271
DV722215	4.7082684	6.286053239	1.577784839	0.000100574	0.07711271
TC538999	5.251634416	6.808826043	1.557191627	0.000112895	0.07711271
Ube2i	11.90457203	12.34706984	0.442497806	0.00013949	0.07711271
BF542749	10.74023303	11.68091656	0.940683533	0.000154434	0.07711271
Ext2_predicted	10.8894966	11.22399898	0.334502379	0.000161949	0.07711271
RGD1311752_predicted	8.703882692	10.44148626	1.737603564	0.000169954	0.07711271
TC522315	2.79964542	4.101760357	1.302114937	0.000179121	0.07711271
AA942848	8.500936128	9.102749214	0.601813086	0.000182133	0.07711271
RGD1308031	5.736834927	6.676339719	0.939504792	0.000186685	0.07711271
Raph1_predicted	6.652550515	9.501294881	2.848744366	0.000196584	0.07711271
Col5a3	9.457522664	10.38121689	0.923694229	0.000206023	0.07711271
BF523017	10.85115842	11.3588647	0.507706279	0.000206202	0.07711271
CB547155	9.262431376	9.602202763	0.339771388	0.000218209	0.07711271

Pcsk3	9.563934918	10.34288495	0.778950036	0.000220488	0.07711271
TC545808	8.730436041	9.609236409	0.878800369	0.000220793	0.07711271
Mfap5_predicted	5.818035999	7.453695841	1.635659842	0.000225576	0.07711271
LOC684302	9.817000952	10.28928105	0.472280097	0.000228335	0.07711271
BE111891	1.08420639	1.983656252	0.899449863	0.000230407	0.07711271
Adam3	1.795552474	4.569825212	2.774272738	0.00024164	0.07711271
RGD1309534	8.024579741	8.473932865	0.449353124	0.00025109	0.07711271
XM_224155	1.757920019	4.374588989	2.61666897	0.000254712	0.07711271
Anks1_predicted	4.785255527	5.643215295	0.857959768	0.00025571	0.07711271
TC564875	9.056765795	9.929394592	0.872628797	0.000260742	0.07711271
Zfand2b	10.84791412	11.55774178	0.709827653	0.000270732	0.07711271
CA333998	11.83201426	12.28062694	0.448612678	0.000284527	0.07711271
Mfng	7.749259513	8.266369008	0.517109494	0.000296828	0.07711271
Bambi	8.812389606	9.914938916	1.10254931	0.000302915	0.07711271
XM_346066	1.076776976	2.82468633	1.747909353	0.000311005	0.07711271
Spg21	11.23012332	11.55721656	0.327093237	0.000318552	0.07711271
CO387496	4.453414129	6.536747702	2.083333573	0.000333125	0.07711271
TC554267	8.373468927	9.14689548	0.773426553	0.000346481	0.07711271
Dcn	5.529606864	8.465423531	2.935816667	0.000347064	0.07711271
RGD1309062	6.563975115	8.064163786	1.500188671	0.000352004	0.07711271
Gprk6	12.47356751	13.45420856	0.980641054	0.000358039	0.07711271
RGD1305243_predicted	7.220436673	7.549994374	0.329557701	0.000358398	0.07711271
DV716578	8.728614931	9.434993476	0.706378545	0.00036259	0.07711271
Lipl3_predicted	4.392258287	6.178702783	1.786444496	0.000363005	0.07711271
Mmp24	15.9433976	16.61068899	0.667291392	0.000369777	0.07711271
Tmem77	9.818583769	10.37533496	0.55675119	0.000374996	0.07711271
Ard1_predicted	11.53917153	12.08142809	0.542256562	0.000383345	0.07711271
RGD1566292_predicted	11.21332683	11.62680095	0.413474114	0.000388946	0.07711271
RGD1311331_predicted	7.875456813	8.872995293	0.997538481	0.000400441	0.07711271
BF556192	12.80155585	13.55394357	0.752387717	0.000416696	0.07711271
Ulk1_mapped	8.808173483	9.951782485	1.143609002	0.000418969	0.07711271
XM_341055	6.720993554	7.689316998	0.968323445	0.000434274	0.07711271
Pxn	7.025381997	7.696209005	0.670827008	0.000443884	0.07711271
Prdm7_predicted	8.455624612	9.496578258	1.040953646	0.000449472	0.07711271
Sdcbp2	3.853370711	4.661073334	0.807702623	0.000449476	0.07711271
Nkiras2_predicted	10.80772401	12.34366608	1.535942062	0.000453754	0.07711271
RGD1306939	5.039293136	5.763551483	0.724258347	0.000460339	0.07711271
BF549650	10.67675061	11.62149087	0.944740258	0.000464965	0.07711271
Tcte3_predicted	1.596662365	4.522208185	2.92554582	0.000467652	0.07711271
Rhebl1	12.07802518	12.52188936	0.443864183	0.000468091	0.07711271
RGD1565763_predicted	3.722812164	5.448624313	1.72581215	0.000488696	0.07711271
Setmar	7.220618123	8.453991365	1.233373242	0.000490118	0.07711271
Vwa1	6.169798444	6.711947958	0.542149513	0.000494053	0.07711271
Limk1	13.40359822	14.23252143	0.82892321	0.000495236	0.07711271
Adam24_predicted	1.398107927	3.625631203	2.227523276	0.00049853	0.07711271
DV720603	12.79953832	13.65543137	0.855893047	0.000504802	0.07711271
XM_215391	5.439407969	6.039017881	0.599609912	0.000510659	0.07711271
LOC498368	8.728806947	9.512459784	0.783652838	0.0005142	0.07711271
Zcwpw1_predicted	8.549635796	9.0135438	0.463908005	0.000519295	0.07711271
RGD1306839_predicted	13.69986612	14.17485686	0.474990743	0.000521826	0.07711271
Itga1	8.054333478	8.953630387	0.899296909	0.000524578	0.07711271
AI136665	2.376789645	3.389671123	1.012881477	0.000524619	0.07711271
Pcdh19_predicted	13.08120857	13.962638	0.881429428	0.000533667	0.07711271
BF564180	5.808806457	6.675428768	0.866622311	0.000536071	0.07711271
TC543571	1.274446722	2.996226578	1.721779856	0.000539336	0.07711271
Atp1b3	12.99375325	13.39955207	0.405798822	0.000540772	0.07711271
Kctd10	13.95844395	14.54894225	0.590498302	0.000541089	0.07711271
RGD1565709_predicted	2.770943739	4.053574585	1.282630846	0.000544688	0.07711271
MGC108896	9.927683288	10.78529474	0.857611447	0.00054487	0.07711271
Kctd12_predicted	5.830704506	6.729043191	0.898338685	0.0005464	0.07711271
TC544355	5.942384994	6.836536724	0.894151731	0.000547615	0.07711271
Reps1_predicted	12.54940511	12.97880545	0.429400346	0.000564254	0.077922785
Serpini2	3.851552344	5.63331535	1.781763007	0.000567829	0.077922785

RGD1559697_predicted	7.915361188	8.797601241	0.882240053	0.000592885	0.077922785
RGD1311517	9.050275883	9.381635441	0.331359557	0.000597652	0.077922785
Mobkl2b_predicted	6.180803886	6.909643322	0.728839436	0.00059883	0.077922785
Emp2	6.804079694	7.619840368	0.815760674	0.000601579	0.077922785
Ralgds	11.79767933	12.51812827	0.72044894	0.000605254	0.077922785
AY387076	1.468185811	3.89156566	2.423379849	0.00061619	0.077922785
AA799294	11.50558544	12.18395453	0.678369094	0.000618851	0.077922785
Brms1	11.25655293	12.15467322	0.898120294	0.000619669	0.077922785
Ankrd39_predicted	10.36845557	10.96848401	0.600028443	0.000637893	0.07819106
AI136427	6.037482625	7.335216483	1.297733859	0.000644122	0.07819106
Gjb3	3.57422167	5.651481798	2.077260129	0.000644829	0.07819106
AI501306	3.777011055	5.108158861	1.331147806	0.000653803	0.07819106
AW918392	11.51957268	12.3825602	0.862987521	0.000659521	0.07819106
Nmnat3	5.564449892	6.751676458	1.187226566	0.000661028	0.07819106
CB546810	5.801761173	6.857025975	1.055264802	0.000661036	0.07819106
XM_341150	7.490252385	7.995806521	0.505554136	0.000662575	0.07819106
ENSRNOT00000049215	5.487755572	6.491754516	1.003998945	0.000677502	0.07819106
Sat2_predicted	11.41485986	12.07821741	0.663357553	0.000679078	0.07819106
Sult5a1_predicted	4.028117727	5.018790107	0.99067238	0.000684007	0.07819106
Lifr	4.766105414	5.676759972	0.910654558	0.00068905	0.07819106
Sbk1	7.24921258	8.326023234	1.076810655	0.000691331	0.07819106
AW920693	5.251069432	6.325335641	1.074266209	0.000708009	0.078249597
Bag1_predicted	12.05780626	12.62124582	0.56343956	0.000709335	0.078249597
RGD1565253_predicted	6.764567091	7.373216758	0.608649667	0.000742443	0.07936277
Entpd1	4.234569182	5.684165794	1.449596612	0.000749336	0.07936277
RGD1564089_predicted	8.457456075	8.924711958	0.467255883	0.000750321	0.07936277
Apom	7.669673822	8.452252945	0.782579124	0.000753429	0.07936277
LOC682679	10.40772142	11.04564296	0.63792154	0.000755786	0.07936277
RGD1307465	4.52394686	5.029958008	0.506011148	0.000759693	0.079499818
RGD1560410_predicted	9.611168666	10.1887279	0.577559237	0.000766138	0.079628835
TC523645	7.803548588	8.242734021	0.439185432	0.000774025	0.080124927
TC539202	11.55544897	11.94668132	0.391232348	0.000783413	0.080601813
Centg3_predicted	12.84619462	13.9344075	1.088212888	0.000803447	0.081294469
RGD1311747_predicted	14.02434855	14.43900737	0.414658825	0.000825545	0.082438477
Shank1	10.5815021	11.58272927	1.001227168	0.000834894	0.082830708
Tacstd2	4.848974747	7.909738294	3.060763548	0.000838122	0.082831536
Tm6p1	11.90498256	12.32688801	0.421905453	0.000842078	0.082831536
Epb4.1l4a_predicted	7.708633228	8.084206681	0.375573454	0.000855828	0.082831536
TC539235	12.57769919	12.98429461	0.40659542	0.000857171	0.082831536
Slc14a2	4.278113831	5.499582517	1.221468686	0.000862335	0.082831536
XM_233669	11.02643522	11.3979456	0.371510374	0.000863122	0.082831536
Zcrb1	11.54763267	11.88828262	0.340649953	0.000871627	0.082831536
TC537987	10.44925387	11.35946001	0.910206148	0.000882803	0.083021035
Txnl5_predicted	12.08651224	12.77338822	0.686875981	0.000891118	0.083021035
Mc4r	4.776734293	6.357261766	1.580527472	0.000907649	0.083047388
Mapkapk2	12.24825701	13.41044477	1.162187759	0.000920767	0.083047388
Gpr113_predicted	2.71239918	4.350850097	1.638450917	0.000930512	0.083047388
RGD1563440_predicted	7.495317587	7.96665657	0.471338983	0.000939173	0.083047388
RGD1311484	7.4650719	8.873662257	1.408590356	0.000941116	0.083047388
Kremen1	3.748222464	5.539540629	1.791318165	0.000944467	0.083047388
Ppa2_predicted	14.6818083	15.0607799	0.378971597	0.000954418	0.083047388
Sirt7_predicted	11.76272884	12.11246965	0.349740806	0.000958533	0.083047388
TC562299	6.335779103	6.981390852	0.645611749	0.00096025	0.083047388
TC556681	11.48157966	12.18693567	0.70535601	0.000961791	0.083047388
Rasd2	9.911490969	11.62097176	1.709480787	0.000964146	0.083047388
Hsdl1	9.808903868	10.18720536	0.378301493	0.000966111	0.083047388
LOC500377	5.693389243	8.157926491	2.464537247	0.000966231	0.083047388
Dhrs4	7.792333232	8.478215325	0.685882094	0.000966303	0.083047388
TC562439	6.699930546	8.249882838	1.549952292	0.00097222	0.083047388
Scamp3	11.23002496	11.81910346	0.589078502	0.000984701	0.083582002

### H2A.Z hypervariant depletion changes synaptic protein expression

**Figure 3.**
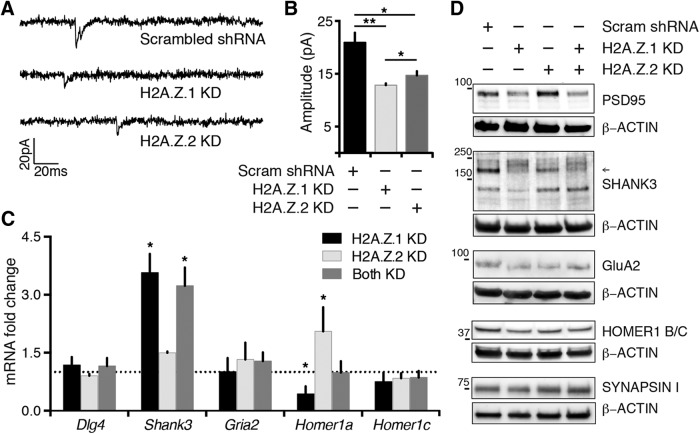
H2A.Z hypervariant-specific RNAi has synaptic effects. ***A***, ***B***, Amplitude of mEPSCs decrease after H2A.Z.1 or H2A.Z.2 depletion. Shown are example traces in ***A*** with quantification of mEPSC amplitude is in ***B*** (*N* = 3; *n* = 15 neurons for Sc, 10 for H2A.Z.1 KD, and 8 for H2A.Z.2 KD; *p < 0.05 and **p < 0.001). Neurons were infected for 5–7 d. ***C***, mRNA levels of indicated genes, normalized to *Gapdh*, represented as fold change of their abundance in control neurons (broken line at 1 on *y*-axis). *N* = 3–5. *p < 0.05. ***D***, Representative Western blottings of synaptic fraction from control or H2A.Z hypervariant-depleted neurons as indicated. Loading control (β-ACTIN) for individual blots are provided and molecular weights of the nearest protein ladder band is indicated. Arrow indicates quantified SHANK3 band (see text). Neurons were infected for 7 d. *N* = 3. Note: levels of synapsin, a presynaptic molecule, remain unaltered after hypervariant depletions.

Because glutamatergic synaptic function was indicated as a hypervariant-specific GO term in the above analysis, and to test whether H2A.Z hypervariant depletions have any impact on the physiologic health of neurons, we performed electrophysiological analyses of membrane and excitatory synaptic properties. Resting membrane potentials and input resistances were not significantly different between control and H2A.Z.1- or H2A.Z.2-depleted neurons. Presynaptic function and/or synaptic density, as inferred by mEPSC frequency, was also not significantly different between conditions (mEPSC frequency per 3 s for control, H2A.Z.1-, and H2A.Z.2-depleted neurons were 0.31 ± 0.31, 0.14 ± 0.06, and 0.13 ± 0.07, respectively). However, mEPSC amplitude, typically indicative of postsynaptic function, was significantly diminished in neurons depleted of either H2A.Z hypervariant (Fig. 3*A*,*B*). To better understand mechanisms of such mEPSC amplitude reduction in H2A.Z hypervariant-depleted neurons, we assessed mRNA and protein levels of several postsynaptic molecules, including many from our microarray screen. A few interesting cases representing diverse effects of hypervariant depletion are presented in Figure 3*C*,*D*.

A contrasting effect of hypervariant depletion was noted in two instances: postsynaptic density protein 95 (PSD-95) and SHANK3. PSD-95 is the anchoring protein encoded by *Dlg4* and found exclusively in the postsynaptic density, while SHANK3 is another member of the postsynaptic density that is encoded by the high-confidence autism candidate gene *Shank3*. These two gene products were strongly reduced in neurons depleted of H2A.Z.1, but not H2A.Z.2 (Fig. 3*D*). Compared to control, PSD-95 band intensities were 48.58 ± 12.4%, 102.34 ± 26.72%, and 65.95 ± 17.42% in H2A.Z.1, H2A.Z.2, and both KD, respectively (*N* = 3). Such an effect of H2A.Z.1 KD on PSD-95 must be indirect (not regulated at the gene transcription level), as *Dlg4* mRNA levels remained unchanged in H2A.Z.1-depleted neurons (Fig. 3*C*). For SHANK3, compared to control, band intensities were 20.25 ± 7.6%, 47.74 ± 20.35%, and 15.34 ± 12.6% in H2A.Z.1, H2A.Z.2, and both KD, respectively (*N* = 3). However, the effect on SHANK3 is interesting, as *Shank3* mRNA levels are upregulated specifically in response to H2A.Z.1 depletion, perhaps as a response to cellular feedback pathways triggered by loss of the protein.

We also tested levels of the ligand-gated ion channel GluA2 (AMPA receptor; coded by *Gria2*, also called GluR2) and postsynaptic density protein HOMER1 (coded by *Homer1*) after H2A.Z hypervariant depletion. As shown in Figure 3*D*, we observe no notable difference in signal of either GluA2 or HOMER1B/C after H2A.Z.1 or H2A.Z.2 knockdown. However, the activity-induced short *Homer1a* mRNA isoform was downregulated by H2A.Z.1 loss and upregulated by H2A.Z.2 depletion (Fig. 3*C*). Further studies are required to confirm if such effects are direct influences of H2A.Z hypervariants on *Homer1a* transcription.

Together, we find that depletion of both H2A.Z.1 and H2A.Z.2 leads to decreased amplitude of mEPSCs, which may be explained by altered expression of postsynaptic proteins. The effects of H2A.Z.1 and H2A.Z.2 knockdown on the tested synaptic proteins, however, are apparently mediated by transcription-independent means in most cases given the lack of change at the pre-mRNA level.

### H2A.Z.1 and H2A.Z.2 depletion differentially impact *Arc/Arg3.1* transcription

**Figure 4.**
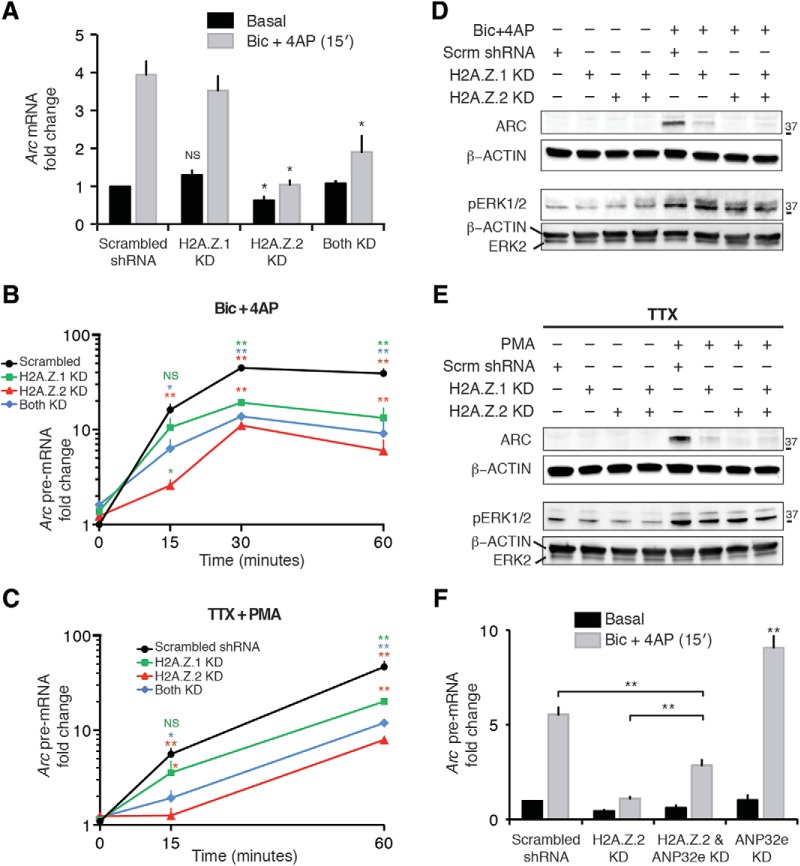
H2A.Z hypervariant-specific RNAi has differential effects on activity-induced *Arc* transcription. ***A***, Graphical representation of *Arc* mRNA level at 15 min after treatment with Bic + 4AP in indicated neuronal groups as detected by qPCR and normalized to *Gapdh*. *N* = 3–4 for each group; *p < 0.05. NS, not significant. ***B***, ***C***, Representation of *Arc* pre-mRNA level at different time points after treatment with Bic + 4AP (***B***) or TTX + PMA (***C***) in indicated groups of neurons (5–6 d postinfection) as detected by qPCR and normalized to *Gapdh*. *N* = 3–4 for each time point; *p < 0.05 and **p < 0.01. NS, not significant. Two sets of data in statistical comparison are represented by the position and the color of asterisk(s) or a NS. For example, in ***B***, the green * on top of the 15 min H2A.Z.2 KD time point (red triangle) represents a statistical difference between the means of H2A.Z.2 and H2A.Z.1 KD group (green square) at the same time point. ***D***, ***E***, Representative Western blottings of whole-cell lysate from neurons infected as indicated for 5–6 d. Neurons were treated with Bic + 4AP (***D***) or TTX + PMA (***E***) for either 60 min to detect ARC, or for 15 min to detect phosphorylated ERK1/2. ***F***, Representation of *Arc* pre-mRNA level at 15 min after treatment with Bic + 4AP in indicated neuronal groups as detected by qPCR and normalized to *Gapdh*. *N* = 3; **p < 0.01.

To study direct roles of H2A.Z hypervariants on transcriptional regulation of a model gene, we chose to study the IEG *Arc/Arg3.1* (referred to as *Arc* from here on), which responds rapidly to neuronal activity (Guzowski et al., 1999; Saha et al., 2011). *Arc* was chosen for several reasons: (1) it is a neuron-specific IEG, which has been extensively studied previously by us and others, (2) its promoter and transcriptional start site (TSS) regions are enriched with H2A.Z-containing nucleosomes (our unpublished observations), and (3) it is one of the genes recently shown to be regulated by H2A.Z in cognitive functions (Zovkic et al., 2014). Neuronal activity was stimulated in dissociated neurons by GABA_A_ receptor antagonist (Bic)-induced disinhibition and K^+^ channel blocker (4AP)-induced increased burst frequency (Bic + 4AP; Papadia et al., 2005). Using the Bic + 4AP protocol, we induced gene transcription and evaluated *Arc* mRNA abundance at 15 min after treatment. Basal and induced *Arc* mRNA levels were unaffected by H2A.Z.1 depletion but were significantly diminished in H2A.Z.2-depleted cells (Fig. 4*A*).

Because mRNA abundance is not necessarily a reflection of transcription per se but rather the net result of both synthesis and decay rates, we next evaluated the direct *Arc* transcription product: its pre-mRNA (Wu and Brewer, 2012). We detected no significant differences in the basal level of *Arc* pre-mRNA with H2A.Z hypervariant depletions (Fig. 4*B*, 0-min time point). After Bic + 4AP treatment, *Arc* pre-mRNA levels increased significantly within 15 min and continued to increase with time in control neurons. However, in agreement with the mRNA data (Fig. 4*A*), the pre-mRNA response in H2A.Z.2-depleted neurons was attenuated at the early time point (15 min) and was also significantly reduced at later points (Fig. 4*B*). In contrast, H2A.Z.1 depletion had no effect on *Arc* pre-mRNA levels at 15 min. However, at later time points, H2A.Z.1-depleted neurons had significantly less *Arc* pre-mRNA than in control neurons and yet had significantly more than in neurons depleted of H2A.Z.2 (Fig. 4*B*). These results suggest that although the loss of H2A.Z.1 impaired *Arc* transcription significantly at later time points, loss of H2A.Z.2 had a much stronger effect at all time points. At the protein level, hypervariant-specific effects on activity-induced ARC abundance were generally reflective of *Arc* pre-mRNA expression patterns (Fig. 4*D*). When both hypervariants were depleted, pre-mRNA results were not significantly greater than H2A.Z.2 depletion alone, and were in fact intermediate between the two hypervariants. While trying to knockdown both H2A.Z.1 and H2A.Z.2, we had to titer our viral infection and infect with less of each virus kind to avoid causing viral overload-induced cell death. Therefore, less knockdown was achieved in the dual KD cells compared to their single KD counterparts. Such reduced knockdown may explain the intermediate effect of knocking down both H2A.Z.1 and H2A.Z.2 on *Arc* transcription.

Because some synaptic alterations were noted due to H2A.Z hypervariant depletion and because Bic + 4AP treatment is a synapse-dependent activity induction protocol, we next tried to induce *Arc* nonsynaptically by jump-starting the MAPK pathway, otherwise employed by activity-induced gene transcription signals (Rosen et al., 1994; Wiegert and Bading, 2011). The MAPK pathway was extrasynaptically activated via PKC using PMA (Schultz et al., 1997) in the presence of TTX to prevent any extracellular input via neuronal activity, and this treatment induced rapid transcription of *Arc* (Fig. 4*C*). When H2A.Z hypervariant-depleted neurons were stimulated using this protocol, the outcome was similar to what was seen with the Bic + 4AP protocol, both at the pre-mRNA and protein levels (Fig. 4*C*,*E*). These observations suggest that consequences of H2A.Z hypervariant depletion on activity-induced *Arc* transcription are most likely not limited to effects of changing synaptic components. Supporting this assertion is the observation that H2A.Z hypervariant depletion had no effect on activity-induced activation of the MAPK pathway as indicated by phospho-ERK1/2 levels (Fig. 4*D*,*E*).

To further test the specificity of the H2A.Z.2 depletion-induced phenotype, we attempted to rescue rapid *Arc* induction by combining shRNA-mediated knockdown with coexpression of shRNA-insensitive H2A.Z.2 (Myc-H2A.Z.2-IS). Several such experimental designs were unsuccessful, likely due to lack of regulatory post-translational modifications on exogenous constructs in the absence of endogenous proteins and/or perturbation of a critical balance of endogenous levels of these hypervariants and their chaperones. The latter possibility is based on a recent study where it was demonstrated that phenotype loss due to depletion of ANP32E, an H2A.Z chaperone that removes nucleosomal H2A.Z, could be rescued by codepletion of ANP32E and H2A.Z (Alatwi and Downs, 2015). Accordingly, we codepleted ANP32E (92.0 ± 0.1% knockdown on day 5 after infection) with H2A.Z.2 and observed a partial rescue of rapid *Arc* transcription (Fig. 4*F*). Taken together, our data show nonredundant roles of H2A.Z hypervariants in the regulation of *Arc* transcription and hint at the importance of a fine balance between histones and their chaperones in mediating such responses.

### H2A.Z hypervariants are present near the *Arc* TSS and regulate its transcription

**Figure 5.**
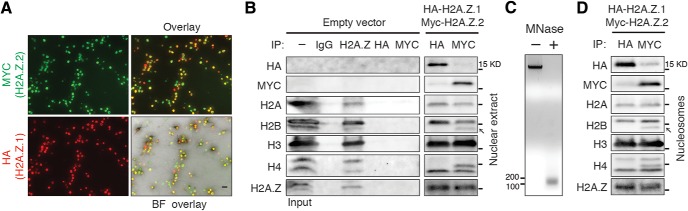
Distinct properties of HA-H2A.Z.1- and MYC-H2A.Z.2-containing nucleosomes. ***A***, Representative image of immunocytochemical analysis of HA-H2A.Z.1 and MYC-H2A.Z.2 expression in unstimulated neurons. Approximately 70% neurons expressed both hypervariants. Scale bar, 20 μm. ***B***, Co-IP assay from nuclear lysate preparation of neurons exogenously expressing a control vector or HA-H2A.Z.1 and MYC-H2A.Z.2. *N* = 3. ***C***, Representative image of genomic DNA obtained from untreated or MNase-treated nuclei of neurons treated as described above. The band size of ∼150 bp indicates that MNase digestion predominantly produced mononucleosomes. *N* = 3. ***D***, Co-IP assay from single nucleosome preparation of neurons exogenously expressing HA-H2A.Z.1 and MYC-H2A.Z.2. Antigen-antibody complex was resolved by electrophoresis and blotted for indicated histones. The 15-kDa protein ladder band is indicated in all panels. Arrow indicates unique band found only in anti-MYC precipitate. *N* = 3.

H2A.Z is enriched near *Arc* TSS (Zovkic et al., 2014). To identify specific hypervariant(s) in this enrichment, we exogenously expressed distinctly tagged hypervariants (HA-H2A.Z.1 and MYC-H2A.Z.2) in neurons and probed for their expression using antibodies against the HA- or MYC-epitope tags (Fig. 5*A*). Usually, protein overexpression is concerning, because it may cause, among other things, artifacts that include ectopic subcellular localizations and inaccurate protein complex formations. Despite such limitations, we reasoned that increased dosage of tagged-H2A.Z hypervariants in the free-floating nucleoplasmic pool may not have any negative consequences as H2A.Z incorporation into its functionally relevant complex (nucleosomes) is tightly regulated by chaperone-dependent mechanisms (Billon and Cote, 2013), and so this overexpression will not necessarily have unwanted nucleosomal incorporation of H2A.Z despite its artificial abundance.

To verify nucleosomal incorporation of tagged-H2A.Z hypervariants, we performed co-IP assays. These assays, with anti-IgG, anti-H2A.Z, anti-HA and anti-MYC from nuclear lysates, or anti-HA and anti-MYC from mono-nucleosome preparations (Fig. 5*B*–*D*), detected stable association of both hypervariants with other nucleosomal histones H2B, H2A, H3 and H4 suggesting that tagged-H2A.Z molecules are incorporated into nucleosomes. Interestingly, (1) HA and MYC IPs probed for their respective hypervariants revealed very little association between HA-H2A.Z.1 and MYC-H2A.Z.2, suggesting that very few H2A.Z nucleosomes contain both hypervariants; and (2) we detected a unique band in H2B, presumably representing an unidentified post-translationally modified state of H2B, in the precipitate with MYC-H2A.Z.2, but not HA-H2A.Z.1 (Fig. 5*B*,*D*). These data suggest that certain H2A.Z.2-containing nucleosomes may have unique post-translational signatures and possibly unique functions.

**Figure 6.**
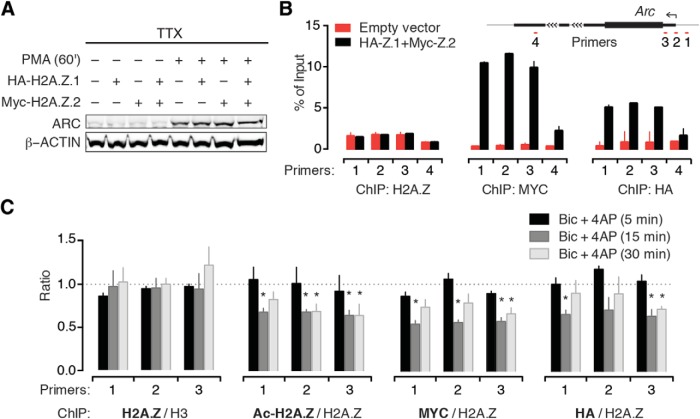
Both H2A.Z hypervariants are present near the *Arc* TSS and mediate its transcription. ***A***, Representative Western blotting of whole-cell lysate from neurons infected as indicated for 5–6 d. Neurons were treated with TTX + PMA for 60 min to assess ARC expression. *N* = 3. ***B***, top, Graphical map (not to scale) depicting relative position of ChIP primers near *Arc* TSS. Bottom, Quantification of H2A.Z, MYC-H2A.Z.2, and HA-H2A.Z.1 binding to the *Arc* TSS region in untreated neurons as determined by ChIP with antibodies against H2A.Z, MYC, and HA epitopes. *N* = 4. ***C***, Quantification of H2A.Z, Ac-H2A.Z, MYC-H2A.Z.2, and HA-H2A.Z.1 binding to *Arc* TSS region in neurons treated with Bic + 4AP for indicated time periods. *N* = 4. Data are normalized as indicated and expressed as fold change compared to untreated samples (denoted by the broken line at 1 on *y*-axis).

To confirm that functional incorporation of tagged-H2A.Z molecules into the *Arc* promoter region did not interfere with transcriptional activity, we expressed HA-H2A.Z.1 and MYC-H2A.Z.2 in mature neurons and induced *Arc* transcription with PMA + TTX. Overexpression of one or both hypervariants did not alter *Arc* transcription at pre-mRNA or protein level (Fig. 6*A*).

Next, we performed ChIP assays to detect H2A.Z and its hypervariants near the *Arc* TSS. We tested several anti-H2A.Z antibodies and found the ChIP grade antibody from Millipore to be most efficient. Using this antibody, we detected significant enrichment of H2A.Z near the *Arc* TSS (primer pair 1, 2, and 3) compared to *Arc* gene body (primer 4; Fig. 6*B*). Primer pairs 1 and 3 roughly represent the −1 and +2 (often referred to as +1) nucleosomes across the *Arc* TSS (Kim et al., 2010), while primer pair 2 denotes a region across the TSS, which instead of being nucleosome-free, houses a nucleosome (+1) in *Arc* (see H3K4.me data in the study by Kim et al., 2010). Like total H2A.Z, both MYC-H2A.Z.2 and HA-H2A.Z.1 were also enriched near the TSS compared to the gene body (Fig. 6*B*). Although strong signals were obtained near the TSS with both anti-HA and anti-MYC ChIP compared to empty vector controls, these signals cannot be directly compared as their apparent difference is likely to be due to dissimilarity in antibody efficiency. For example, this trend is reversed when similar ChIP assays were performed after overexpressing hypervariants with swapped tags (data not shown). Taken together, we conclude that both H2A.Z hypervariants are present near the *Arc* TSS.

To distinguish the potentially different roles of H2A.Z hypervariants in activity-induced *Arc* transcription at the gene level, we treated neurons with Bic + 4AP for various time periods and performed ChIP with anti-H2A.Z, anti-MYC, anti-HA, and anti-acetylated H2A.Z (Ac-H2A.Z) antibodies (Fig. 6*C*). Acetylation of H2A.Z is an important epigenetic modification associated with active or primed promoters (Soboleva et al., 2014). On Bic + 4AP treatment, H3-normalized total H2A.Z levels were unchanged at any of the three nucleosomes across the *Arc* TSS at all time points tested (see Discussion). However, ChIP signal for Ac-H2A.Z drops significantly at all three nucleosomes at 15 min after treatment and remains reduced in the +1 and +2 nucleosomes after 30 min. Although total level of H2A.Z remained unchanged, we detected a significant drop of H2A.Z-normalized MYC-H2A.Z.2 and HA-H2A.Z.1 at the +2 nucleosome 15 and 30 min after treatment, suggesting ongoing activity-induced nucleosomal turnover of H2A.Z (nucleosomal remodeling). Interestingly, at the +1 nucleosome, only MYC-H2A.Z.2 levels dropped significantly at 15 min after treatment, indicating a possible H2A.Z.2-specific role for this nucleosome at this early time point. This inference is corroborated by our data in that the most severe effect on *Arc* induction was found after the depletion of H2A.Z.2 (Fig. 4*B–E*).

### H2A.Z.2 is required for RNA Pol II pausing near the *Arc* TSS

**Figure 7.**
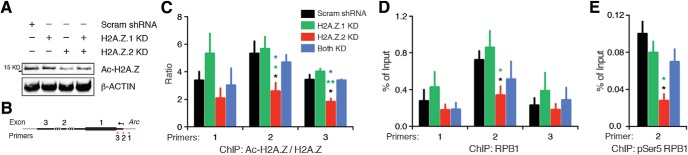
H2A.Z.2 facilitates priming of the *Arc* promoter. ***A***, Representative Western blotting of whole-cell lysate showing Ac-H2A.Z levels in neurons depleted of H2A.Z hypervariants as indicated. *N* = 3. ***B***, Graphical map (not to scale) depicting relative position of ChIP primers near *Arc* TSS. ***C***, Quantification of Ac-H2A.Z (normalized by total H2A.Z) binding to *Arc* TSS region in neurons treated as indicated, determined by ChIP. *N* = 4. Two sets of data in statistical comparison are represented by the position and the color of asterisk(s). ***D***, ChIP data demonstrating binding of RPB1 to the *Arc* TSS region in control neurons or H2A.Z hypervariant-depleted neurons as indicated. *N* = 4. Note the enrichment of RPB1 at the TSS (primer 2) in control neurons (black) indicating promoter-proximal RNA Pol II pausing. ***E***, ChIP data demonstrating binding of pSer5-RPB1 to the *Arc* TSS in control and hypervariant-depleted neurons. *N* = 3. *p < 0.05 and **p < 0.01.

To study the underlying mechanism of H2A.Z.2’s role in rapid *Arc* transcription (Fig. 4), we tested the effect of its knockdown on histone acetylation and promoter proximal RNA Pol II pausing based on recent reports (Saha et al., 2011; Vardabasso et al., 2015). In melanoma cells, knockdown of H2A.Z.2, but not H2A.Z.1, attenuates acetylation of histone H3 and H4 globally (Vardabasso et al., 2015). We observed no such effect of H2A.Z.2 depletion on H3 or H4 acetylation in neurons (data not shown). This effect is presumably a cell-type-specific phenomenon relevant to dividing cells. Instead, we noted that H2A.Z.2 depletion led to a decrease in Ac-H2A.Z levels globally (Fig. 7*A*) and near the *Arc* TSS (Fig. 7*B*,*C*). Note that the data presented in Figure 7*C* are normalized by total H2A.Z, the level of which remained unaltered after hypervariant knockdown. The effect was not mimicked by H2A.Z.1 depletion. This suggests that H2A.Z.2 depletion results in selective loss of acetylation, not total H2A.Z, near the TSS.

Presence of TSS proximal H2A.Z highly correlates with another chief component of rapid IEG transcription, RNA Pol II pausing (Day et al., 2016). We have previously shown that RNA Pol II is loaded and paused near TSS of *Arc* and other rapidly induced IEGs, and underlies the ability of these genes to respond to activity within a few minutes (Saha et al., 2011). Here, we tested whether there is a relation between Pol II pausing and H2A.Z hypervariants at the *Arc* TSS. Using ChIP with an antibody against RPB1 (N-terminus), the largest Pol II subunit, we found that RNA Pol II pausing at the *Arc* TSS is diminished significantly in neurons depleted of H2A.Z.2, but not H2A.Z.1. While it is unlikely that our anti-RPB1 antibody precipitates nonspecific proteins or is less efficient after hypervariant depletion, we sought to corroborate our findings with another antibody to further ensure that H2A.Z.2 depletion was affecting paused Pol II. We performed ChIP with an antibody specific to RPB1 phosphorylated at serine 5 (pSer5) residue of C-terminal domain heptad repeats. pSer5-RPB1 represents the pool of RPB1 that is transcriptionally engaged, but has not entered productive elongation (paused). Our data indicate that actively engaged Pol II at the +1 nucleosome of *Arc* is significantly reduced by the loss of H2A.Z.2, but not H2A.Z.1 (Fig. 7*E*). This hypervariant-specific loss of Pol II pausing provides an explanation for our observations at 15-min post-Bic + 4AP treatment (Fig. 4*B*,*C*), where *Arc* pre-mRNA levels were minimal in H2A.Z.2-, but not in H2A.Z.1-, depleted neurons.

### H2A.Z.1 plays context-dependent roles in *Arc* transcription

**Figure 8.**
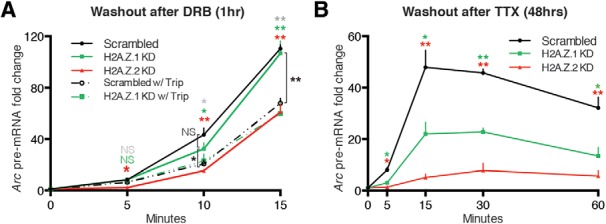
H2A.Z.1 plays context-dependent roles in *Arc* transcription. ***A***, Graphical representation of the level of *Arc* pre-mRNA at different time points after 1-h treatment with DRB followed by its washout for indicated time periods in the presence or absence of Trip in indicated groups of neurons (infected for 5–6 d) as detected by qPCR and normalized to *Rn18s* (18s rRNA; DRB inhibition-insensitive, Pol III-dependent transcription). Gray, green and red asterisk(s) or NS represent statistical comparison between control (Scrambled) responses and responses in Scrambled w/Trip, H2A.Z.1 KD w/Trip and H2A.Z.2 KD, respectively. Connecting brackets delineate other comparisons, shown in black asterisk(s) or NS. ***B***, Similar dataset as in ***A***, except that control neurons or hypervariant-depleted neurons were treated with TTX for 48 h followed by its washout for indicated time periods. Two sets of data in statistical comparison are represented by the position and the color of asterisk(s) or NS. For both ***A***, ***B***, *N* = 4–5 for each time point; *p < 0.05 and **p < 0.01. NS, not significant.

To determine a potential role for H2A.Z.1 in *Arc* transcription, we hypothesized that it may facilitate Pol II recruitment or elongation during active transcription. To test these possibilities, we induced *Arc* transcription using the DRB washout protocol with or without Trip. DRB is a nucleoside analog that inhibits mammalian transcription by interfering with the DRB sensitivity-inducing factor complex that enables RNA Pol II pausing in the presence of negative elongation factor (NELF), and then facilitates productive elongation once NELF falls off in response to external signals (Kwak and Lis, 2013). Because DRB can be washed out, DRB washout assays have been used to achieve rapid and synchronized transcription elongation without additional extranuclear signals (Lee et al., 2015). When control neurons were subjected to this assay, we observed a rapid and robust *Arc* induction (Fig. 8*A*). DRB washout resulted in significant upregulation of *Arc* pre-mRNA levels within three minutes, which then continued to increase with time to reach >100-fold at 15 min. Depletion of H2A.Z.2 resulted in, as we saw with Bic + 4AP and TTX + PMA assays, an attenuated response at the early time point that then partially recovered at later time points while remaining significantly less than the normal response (Fig. 8*A*). In contrast, depletion of H2A.Z.1 had no effect on DRB washout-triggered robust *Arc* transcription (Fig. 8*A*), indicating that H2A.Z.1 is likely not involved in the productive elongation phase.

Next, to test for any role of H2A.Z in RNA Pol II recruitment, a phenomenon that sustains robust responses by providing the gene promoter with a chain of Pol II complexes, we washed out DRB in the presence of Trip, a diterpene triepoxide isolated from natural sources. Trip inhibits Pol II recruitment to promoters by inhibiting TFIIH, the pre-initiation complex component that mediates Pol II binding to DNA (Titov et al., 2011). When DRB washout was performed in the presence of a low concentration of Trip, *Arc* transcription was significantly reduced at later time points (Fig. 8*A*) suggesting that the slightest interference with Pol II recruitment compromises the robustness of the response. Had H2A.Z.1 played a role in Pol II recruitment, its depletion would have had an effect comparable to washout with Trip in control cells. However, that does not seem to be the case as the *Arc* response is significantly different between these two groups (Fig. 8*A*, compare broken black line with green line). Moreover, control and H2A.Z.1-depleted neurons responded similarly to induction in the presence of Trip (Fig. 8*A*, compare broken black and green lines), suggesting that H2A.Z.1 is unlikely to be required for Pol II recruitment.

To test the roles of H2A.Z hypervariants in another neurobiological context, we next performed the TTX washout assay (Rao et al., 2006; Saha et al., 2011). This assay involves treating neurons with TTX for 48 h to induce homeostatic synaptic changes, followed by TTX washout to trigger rapid and robust network activity resulting in *Arc* transcription (Fig. 8*B*). Consistent with other induction assays so far, H2A.Z.2 depletion had an attenuating effect on early transcriptional response, followed by partial recovery at later time points (Fig. 8*B*). Surprisingly, depletion of H2A.Z.1 significantly reduced the *Arc* response throughout, even the early time points. This is in contrast with the insensitivity of the *Arc* response at early time points in all induction assays, including 15-min postinduction with Bic + 4AP under resting (control) conditions. Together, our data suggest that H2A.Z.1 exerts context-dependent effects on activity-induced *Arc* transcription.

### Gene- and context-specific roles of H2A.Z hypervariants in rapid IEG transcription

**Figure 9.**
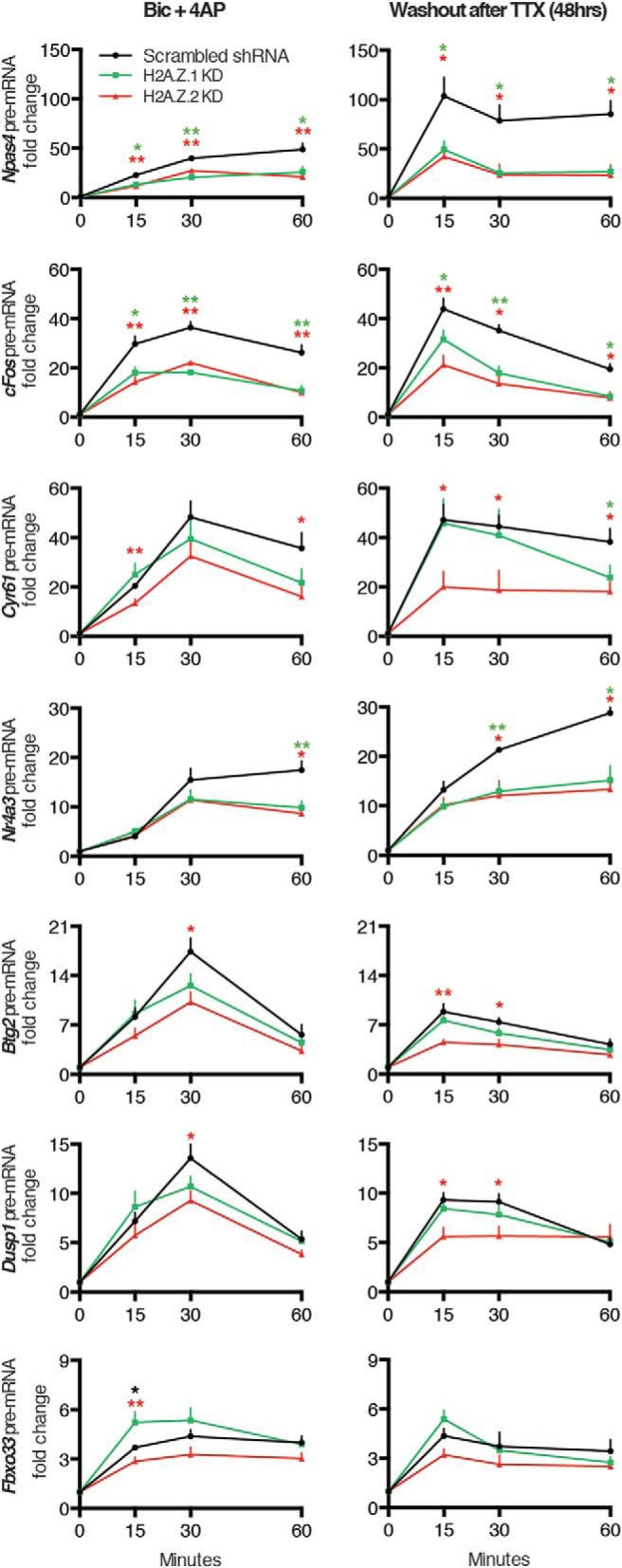
H2A.Z.1 and H2A.Z.2 play context-dependent roles in activity-induced transcription of rapid IEGs. Graphical representation of pre-mRNA levels for noted IEGs at different time points after two treatment regimens. Left, Bic + 4AP treatment. Right, 48-h treatment with TTX followed by its washout for specified time periods in indicated groups of neurons as detected by qPCR and normalized to *Gapdh*. *N* = 4–7 for each time point; *p < 0.05 and **p < 0.01. NS, not significant. Two sets of data in statistical comparison are represented by the position and the color of asterisk(s).

In a previous study, we identified two groups of IEGs classified based on the presence or absence of paused Pol II near the promoter (Saha et al., 2011). We refer to these two groups as rapid IEGs (genes with paused Pol II) and delayed IEGs (genes without paused Pol II; Saha and Dudek, 2013). Because H2A.Z.2 likely regulates *Arc* transcription via RNA Pol II pausing, we next asked if the roles played by these hypervariants are universal across other rapid IEGs. Several rapid IEGs, identified in our previous screen, were then studied under two neurobiological contexts: (1) activity induced in previously untreated neurons (Bic + 4AP assays); and (2) activity induced after homeostatic changes (TTX washout assays). Responses of a few IEGs in control and H2A.Z hypervariant-depleted neurons are shown in Figure 9, and salient inferences drawn from these data are noted in the following paragraph.

All IEGs in Figure 9 respond to activity under both normal and homeostatic conditions. With some genes, such responses are more robust at early time points after 48 h of TTX treatment (*Npas4*, *cFos*, *Cyr61*, etc.). In general though, most rapid IEGs maintained similar temporal dynamics of expression in both conditions; *Btg2* was an exception in that its response peaked at different times under different conditions. In most of these instances, depletion of either H2A.Z.1 or H2A.Z.2 had either no effect or reduced gene transcription. *Fbxo33* was an exception to this observation, with H2A.Z.1 depletion resulting in a significantly stronger transcriptional response at the early time point in the Bic + 4AP condition only. Several genes (*Cyr61*, *Btg2*, and *Dusp1*) resembled *Arc* in that H2A.Z.2 depletion caused the most severe reductions. In contrast to *Arc* and these genes, H2A.Z.1 or H2A.Z.2 knockdown exerted statistically similar effect on several rapid IEGs (*Npas4*, *cFos*, *Nr4a3*, etc.) under both contexts. Interestingly, we noticed that H2A.Z hypervariant depletions induced different outcomes under different contexts in a few cases. For example, H2A.Z.2 depletion did not alter *Btg2* and *Dusp1* responses significantly at the early time point under normal conditions, but impaired them at the same time point after 48 h of TTX. Interestingly, these two genes are insensitive to H2A.Z.1 depletion for both contexts at all tested time points. Collectively, our data in Figure 9 suggest that H2A.Z.1 and H2A.Z.2 do not have generalized roles in rapid IEG transcription; instead, they play gene-specific roles, which may vary further under different neurobiological contexts.

**Figure 10.**
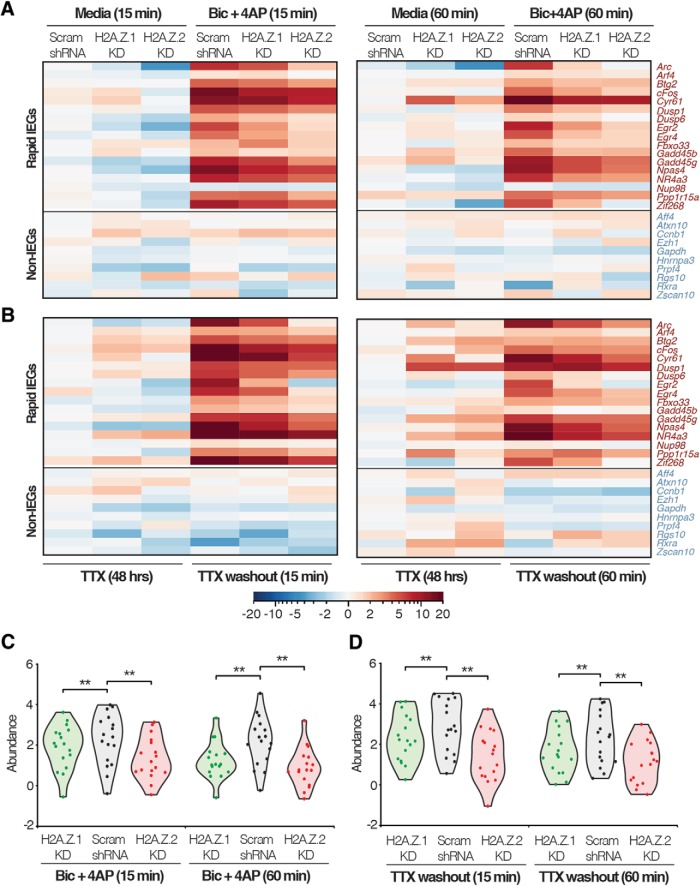
Effect of H2A.Z hypervariant depletion on transcription of rapid IEGs and nonactivity-induced genes. ***A***, Nanostring multiplexed gene expression data (heat maps) representing pre-mRNA levels in control and hypervariant-depleted neurons treated with Bic + 4AP for indicated times. ***B***, Heat maps representing pre-mRNA levels in control and hypervariant-depleted neurons treated TTX for 48 h followed by washout and collected at indicated times. ***C***, ***D***, Violin plots depicting pre-mRNA abundance of all rapid IEGs from hypervariant-depleted neurons compared to that of control neurons treated with Bic + 4AP (***C***) or TTX for 48 h followed by washout (***D***) for indicated times. *N* = 3 for each treatment all time points. *p < 0.05 and **p < 0.01 (Wilcoxon paired nonparametric test).

To further investigate the role of H2A.Z hypervariants in IEG transcription, we conducted NanoString analyses, a multiplexed gene expression analysis system (Geiss et al., 2008), on control and hypervariant-depleted cells under the different neurobiological contexts described above using all IEGs identified in our previous screen (Saha et al., 2011). Figure 10*A*,*B* shows the results for rapid IEGs and non-IEGs (control genes) displayed as heat maps. Notably, results of the NanoString analyses were consistent with our initial qPCR analyses of rapid IEGs in Figure 9. Although there appeared to be gene-specific regulation due to loss of H2A.Z.1 and/or H2A.Z.2, overall comparison of all rapid IEGs tested in control neurons to those tested in hypervariant-depleted neurons suggests that loss of either hypervariant generally resulted in diminished transcription of rapid IEGs at each time point under both neurobiological contexts (Fig. 10*C*,*D*).

### Gene- and context-specific roles of H2A.Z hypervariants in transcription of delayed IEGs

**Figure 11.**
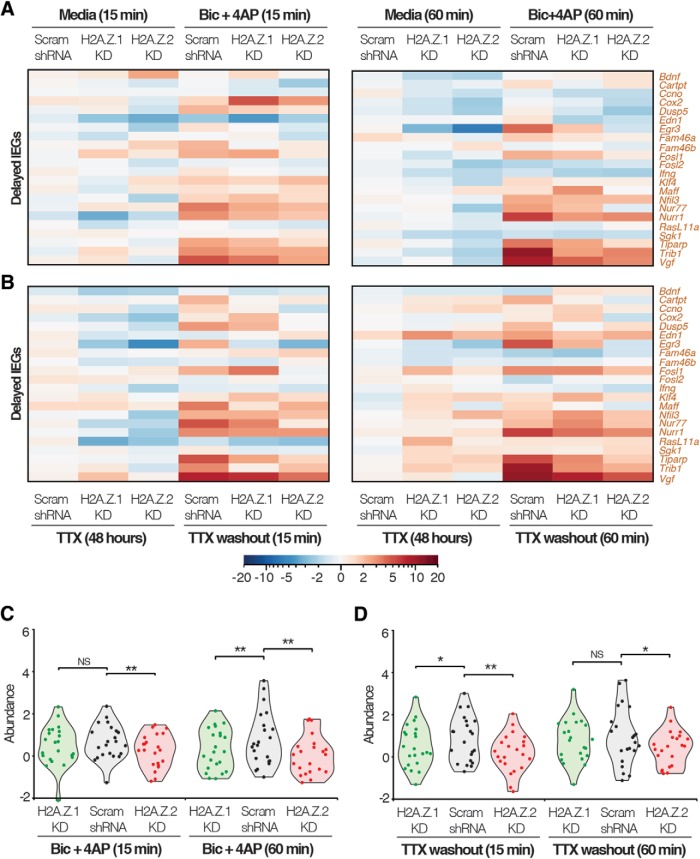
Effect of H2A.Z hypervariant depletion on transcription of delayed IEGs. ***A***, Nanostring multiplexed gene expression data (heat maps) representing pre-mRNA levels in control and hypervariant-depleted neurons treated with Bic + 4AP for indicated times. ***B***, Heat map representing pre-mRNA levels in control and hypervariant-depleted neurons treated TTX for 48 h followed by washout and collected at indicated times. ***C***, ***D***, Violin plots depicting pre-mRNA abundance of all delayed IEGs from hypervariant-depleted neurons compared to that of control neurons treated with Bic + 4AP (***C***) or TTX for 48 h followed by washout (***D***) for indicated times. *N* = 3 for each treatment at all time points. *p < 0.05 and **p < 0.01 (Wilcoxon paired nonparametric test).

Because we saw gene-to-gene variation in H2A.Z hypervariant regulation of rapid IEG transcription, we asked if these hypervariants might also be involved in regulating transcription of delayed IEGs. Based on the lack of a role for paused Pol II in the delayed IEG transcription, we expected to find no effect of hypervariant depletion. However, we found a context-dependent regulation of delayed IEGs, and some trends are noted along the following lines: at 60-min postinduction, depletion of H2A.Z.1 appears to result in downregulation of *Cox2*, *Klf4*, and *Nur77* with Bic + 4AP induction, yet results in upregulation of the same genes on induction after TTX-induced changes. Differential regulation between neurobiological contexts was also evident after loss of H2A.Z.2 in *Cartpt* and *Fam46a* (Fig. 11*A*). Interestingly, group statistical comparisons of delayed IEG responses in hypervariant-depleted neurons to control neurons show that loss of H2A.Z.1 only has a significant effect at the early time point on induction after 48 h of TTX. However, on induction in normal neurons (Bic + 4AP assays), H2A.Z.1 depletion only significantly affects the group at the late time point (Fig. 11*B*). Overall, these data suggest that the roles of H2A.Z hypervariants in regulating transcription of delayed IEGs may vary depending on specific genes and cellular states.

## Discussion

H2A.Z is enriched in nucleosomes near promoters and enhancers of many vertebrate genes and therefore is postulated to play a critical role in gene transcription (Farris et al., 2005; Raisner and Madhani, 2006; Zlatanova and Thakar, 2008). However, roles of the hypervariants H2A.Z.1 and H2A.Z.2 in these processes have often been presumed to be redundant, and have not been categorically questioned (Soboleva et al., 2014). In the current study, we have demonstrated several instances of H2A.Z.1 and H2A.Z.2 nonredundancies: (1) H2A.Z.1 and H2A.Z.2 depletion in cortical neurons affects basal expression of predominantly nonoverlapping sets of genes; (2) depletion of these hypervariants has differential effects on important synaptic molecules like PSD-95 and SHANK3; (3) H2B, perhaps when modified in a certain way, interacts with MYC-H2A.Z.2, but not HA-H2A.Z.1; (4) H2A.Z.1 and H2A.Z.2 depletion differently influence activity-induced *Arc* transcription kinetics; (5) in normal neurons, H2A.Z.2, but not H2A.Z.1, is required near the *Arc* TSS for RNA Pol II pausing; (6) activity-induced transcription of certain IEGs is sensitive to depletion of only one of the two hypervariants (e.g., *Btg2* and *Dusp1* response is less after H2A.Z.2, but not H2A.Z.1, knockdown); and (7) certain IEGs appear to be differentially sensitive to one or both hypervariants in a context-dependent manner (*Fbxo33* and *Maff* after H2A.Z.1 depletion, *Fam46a* after loss of H2A.Z.2). Taken together, our data strongly suggest that H2A.Z hypervariants have important, nonredundant roles and/or functional specificity that should be considered for a full understanding of the part played by H2A.Z in gene transcription.

Three lines of evidence inspired our hypothesis about functional specificity of H2A.Z hypervariants. First, several instances have been observed in nature where genes duplicated during evolution encode very similar protein products that have achieved functional diversity. An important example is found in yeast where several paralogous ribosomal proteins are functionally dissimilar to their nearly identical counterparts (Komili et al., 2007; Steffen et al., 2012). Because mechanisms that work are often preserved in nature, we reasoned that the three amino acid differences in these hypervariants might be enough to allow functional diversity. Second, all three variant amino acid residues reside in functionally pertinent regions thereby making them suitable to mediate alternative functionalities. H2A.Z.1^14S^/H2A.Z.2^14A^ is located in the H2A.Z N-terminal tail, which is heavily acetylated and is linked to H2A.Z transcriptional activity (Bruce et al., 2005; Valdés-Mora et al., 2012). H2A.Z.1^38T^/H2A.Z.2^38S^ is located at the edge of the L1 loop, which plays an essential part in intranucleosomal interactions of H2A.Z (Horikoshi et al., 2013). Such intranucleosomal interactions are also facilitated by the unstructured region of H2A.Z C-terminus (Wratting et al., 2012), which houses the H2A.Z.112^128V^/H2A.Z.2^128A^ variation. Third, knockout of H2A.Z.1 results in embryonic lethality in mice (Faast et al., 2001) indicating that H2A.Z.2 cannot compensate for the loss of its paralog. Together, these observations suggest that H2A.Z hypervariants have nonredundant functions that have not yet been adequately explored.

Our study into functional specificity of H2A.Z hypervariants in activity-induced *Arc* transcription revealed a few interesting aspects of H2A.Z biology. Overall, *Arc* transcription requires both H2A.Z.1 and H2A.Z.2. Depletion of either hypervariant had unique effects on *Arc* transcriptional kinetics that were not compensated by the other paralog. In normal previously untreated neurons (i.e., without homeostatic changes induced by silencing), H2A.Z.2, but not H2A.Z.1, was found to be necessary for the rapid phase of activity-induced *Arc* transcription (within 15 min of treatment) by facilitating RNA Pol II promoter proximal pausing (Fig. 7). Pol II pausing near promoters is facilitated, among other factors, by the position of the +1 nucleosome (Kwak and Lis, 2013; Jimeno-González et al., 2015). Although exact mechanisms remain unknown, it is presumed that the +1 nucleosome acts as a physical barrier to the transcriptionally engaged Pol II. In mammals, the presence of H2A.Z in the +1 nucleosome is positively correlated with Pol II pausing (Ku et al., 2012; Day et al., 2016). In our study, considering that (1) H2A.Z.2 is required for Pol II pausing near the *Arc* TSS and (2) Bic + 4AP treatment removes MYC-H2A.Z.2 from the +1 nucleosome (Fig. 6), we suggest that H2A.Z.2-containing nucleosomes play a dual role in mediating activity-induced transcription. In the resting state before activity, the +1 H2A.Z.2-containing nucleosome would maintain a rigid barrier to Pol II, thereby facilitating its pausing. After activity, dynamic turnover of this nucleosome allows Pol II to commence productive elongation. If true, such a dual-role model could fuel further queries about possible activity-induced signals that convert a rigid nucleosomal barrier into a dynamic gate for Pol II productive elongation. Although this model holds true for activity induced under both normal and prolonged TTX treatment-enabled conditions for *Arc*, further investigation into other IEGs revealed the potential for both gene- and context-specific roles of H2A.Z hypervariants in gene transcription. However, future studies will be required to determine if these hypervariants are directly or indirectly involved in the transcription of other IEGs investigated in this study.

One of our observations was puzzling and therefore difficult to interpret: although there was turnover of MYC-H2A.Z.2 in response to acute increases in neuronal activity, the total levels of H2A.Z (as reported by the Millipore anti-H2A.Z antibody) near the *Arc* TSS did not change over the entire duration o*f* testing. This could be due to weak efficiency of the commercial H2A.Z antibody (Fig. 6*B*, compare signals), which was the best anti-H2A.Z ChIP antibody in our hands. We believe that, as has been previously reported (Zovkic et al., 2014) and is also shown by our anti-MYC ChIP data, activity does indeed induce H2A.Z turnover at the *Arc* TSS.

In comparison to the Pol II pausing-related defined role of H2A.Z.2 in *Arc* transcription, the exact role of H2A.Z.1 in the process is less apparent. In normal, unsilenced neurons, lack of H2A.Z.1 impairs only the late segment of activity-induced *Arc* transcription (30 min and later). As suggested by our DRB washout assays, this is most likely via indirect mechanisms (discussed further below). However, after subjecting neurons to homeostatic plasticity-related changes with TTX exposure for 48 h, H2A.Z.1 depletion also impairs early responses of *Arc* transcription. This repurposing of H2A.Z.1 after prolonged lack of activity is likely mediated by a context-dependent newfound ability to facilitate Pol II pausing. Alternatively, these data could also be explained by heterogeneity of nucleosomal composition in the cell population (discussed further below).

Considering that we could not detect strong interactions of tagged H2A.Z.1 and H2A.Z.2 in our co-IPs (Fig. 5), indicating that they likely do not often coexist in the same nucleosome, and yet show H2A.Z.1 and H2A.Z.2 enrichment at the same nucleosomal positions (Fig. 6*B*), it is possible that these *Arc* TSS nucleosomal loci are populated by different H2A.Z hypervariants in different alleles and/or cells in the population. In normal neurons, only the fraction of these alleles and/or cells that have H2A.Z.2, and therefore paused Pol II, responds to activity. After prolonged TTX treatment, additional alleles and/or cells with H2A.Z.1 attain Pol II pausing via unknown mechanisms and facilitate an enhanced response after TTX washout. This explanation aligns with the noticeably more robust expression of *Arc* after prolonged TTX treatment and washout in comparison to disinhibition-induced activity under previously untreated conditions (compare Figs. 4*B*, 8*B*). Also, the effect of H2A.Z.1 depletion on late, not early, *Arc* transcriptional response (Fig. 4*B*,*D*) falls within the scope of this explanation. On induction of activity in resting conditions, H2A.Z.1-containing alleles and/or cells undertake *Arc* transcription in a Pol II pausing-independent “delayed” fashion, as delayed IEGs do (Saha et al., 2011), thereby accounting for impairments at later time points in H2A.Z.1-depleted neurons. These are exciting possibilities that will require further probing.

Additional experiments will also be required to understand the relevance of the H2A.Z chaperone ANP32E in regulating H2A.Z functions (Mao et al., 2014; Obri et al., 2014). In our hands, several traditional “rescue” attempts involving expression of shRNA-insensitive H2A.Z.2 failed to reverse the loss of *Arc* transcription in H2A.Z.2-depleted neurons. However, the phenotype loss could be partially rescued by codepletion of ANP32E. Because ANP32E removes H2A.Z from nucleosomes (Mao et al., 2014; Obri et al., 2014; Gursoy-Yuzugullu et al., 2015), it is likely that the presence of ANP32E in H2A.Z.2-depleted neurons facilitates continued removal of nucleosomal H2A.Z.2 and subsequent fallouts thereof, including loss of Pol II pausing and inability to respond rapidly. However, codepletion of the chaperone along with the histone preserves nucleosomal H2A.Z.2, at least in part, enabling *Arc* to rapidly respond to some extent. A similar precedence of H2A.Z and ANP32E codepletion partially rescuing phenotype loss due to depletion of one of these factors exists in non-neuronal cells (Alatwi and Downs, 2015). Our findings, combined with this previous report, advocate for the need to approach the complex biology of H2A.Z hypervariants and their chaperones together.

**Table 3.** List of DEGs after H2A.Z.1 and H2A.Z.2 KD-part B (downregulated after H2A.Z.1 KD)

symbol	scrambled expression	shh2az.1 expression	log2 fold change	*p* value	fdr
loc287522	6.259898423	3.781938662	—2.477959761	6.60e—06	0.049404732
rgd1566064_predicted	12.81190937	9.362864495	—3.449044872	7.18e—06	0.049404732
tomm34_predicted	9.80058324	8.124036057	—1.676547183	8.08e—06	0.049404732
ck363699	6.61546991	5.78793267	—0.827537241	1.87e—05	0.065026891
bf562307	9.734906301	8.443322223	—1.291584078	2.29e—05	0.065026891
tc553984	10.57601225	9.76833986	—0.807672386	2.34E—05	0.065026891
AI599250	7.713399206	7.087788209	—0.625610997	3.23E—05	0.076745729
BC098774	7.237548308	5.570550667	—1.666997641	4.77E—05	0.07711271
Grik4	9.223661505	8.523675197	—0.699986308	5.03E—05	0.07711271
Sdhd	14.37797406	13.56563435	—0.812339706	6.16E—05	0.07711271
Napg	10.37368659	9.853267401	—0.520419193	6.42E—05	0.07711271
Mrrf	7.127263566	6.065031172	—1.062232394	8.17E—05	0.07711271
BC091405	10.38125201	9.282285641	—1.09896637	8.74E—05	0.07711271
Tarbp2	12.45655726	12.07159614	—0.384961117	9.39E—05	0.07711271
RGD1309546_predicted	8.534002377	7.502899404	—1.031102973	0.000106301	0.07711271
Grin2c	8.899255961	6.97904754	—1.920208421	0.000106996	0.07711271
TC566987	6.659279818	5.827413166	—0.831866652	0.000108607	0.07711271
TC552897	5.933248175	4.801232308	—1.132015867	0.00010879	0.07711271
Prkar2a	6.163801451	5.313528282	—0.850273169	0.000111853	0.07711271
Glt1d1_predicted	5.121800999	4.103526035	—1.018274964	0.000112311	0.07711271
BF555161	10.59364955	9.470574079	—1.123075469	0.000117617	0.07711271
TC538830	12.53146816	11.24170839	—1.289759763	0.000121745	0.07711271
RGD1566054_predicted	9.115400025	8.101685773	—1.013714251	0.000122074	0.07711271
Lrp11_predicted	13.07487226	11.70424527	—1.370626989	0.000124681	0.07711271
TC536024	7.13278452	6.240473074	—0.892311445	0.000125993	0.07711271
Cited2	10.85269214	8.990111494	—1.862580645	0.000136342	0.07711271
BF397813	7.19066408	5.792890555	—1.397773525	0.000144061	0.07711271
AI112975	6.352713186	5.377218263	—0.975494923	0.000146482	0.07711271
RGD1566367_predicted	5.6358019	5.240674268	—0.395127632	0.00014795	0.07711271
Polr3h_predicted	11.44293632	10.23386016	—1.209076162	0.000150598	0.07711271
Pygb	12.45213506	11.79157662	—0.660558447	0.000154638	0.07711271
AW142634	8.922013872	7.829003261	—1.093010611	0.000156724	0.07711271
BF565628	10.29869369	9.349174788	—0.949518899	0.000157086	0.07711271
Gdap1_predicted	8.365334903	6.792027238	—1.573307664	0.000164033	0.07711271
Ryr2	8.079272174	7.27621553	—0.803056644	0.000164158	0.07711271
BF555945	7.644955812	5.93488388	—1.710071931	0.000166291	0.07711271
Chchd1_predicted	13.92529609	13.52993193	—0.395364161	0.000185585	0.07711271
CB718612	8.141380063	7.317726555	—0.823653508	0.00018998	0.07711271
BF401583	3.807342642	3.135350749	—0.671991893	0.000196042	0.07711271
DV718172	13.52733676	12.61872235	—0.908614414	0.000205766	0.07711271
AW921183	10.34179328	9.467173785	—0.874619492	0.000208601	0.07711271
Atp6v1a1_predicted	10.42042831	10.06973958	—0.350688726	0.000209702	0.07711271
Hexim2_predicted	9.914767395	9.287364476	—0.627402919	0.000237955	0.07711271
Pigh_predicted	6.450865425	5.643206561	—0.807658864	0.000246319	0.07711271
LOC307783	5.032882194	3.665875684	—1.367006509	0.000248578	0.07711271
LOC690163	8.287898848	6.848782046	—1.439116802	0.000252011	0.07711271
RGD1566086_predicted	6.787042716	6.316220869	—0.470821846	0.000252689	0.07711271
TC525615	11.81161754	10.5813283	—1.230289236	0.000256806	0.07711271
Pou3f1	10.95885519	9.374839483	—1.584015704	0.000261757	0.07711271
AW920545	8.612227937	8.006194471	—0.606033466	0.000263301	0.07711271
TC530561	6.361469677	4.977425946	—1.38404373	0.000266017	0.07711271
Vps45	11.18560958	10.64488764	—0.540721933	0.000266506	0.07711271
RGD1565289_predicted	13.51664684	12.46921634	—1.047430504	0.000283049	0.07711271
Klhl20_predicted	8.808515699	7.624582956	—1.183932742	0.000283307	0.07711271
Pdzk8_predicted	8.242171572	7.347607077	—0.894564495	0.000305242	0.07711271
TC560958	11.93185862	11.11468764	—0.817170979	0.000305894	0.07711271
Cops7a_predicted	13.1106929	12.4375094	—0.673183498	0.000306494	0.07711271
TC528997	9.156193399	8.115543858	—1.040649541	0.000308314	0.07711271
RGD1310351_predicted	10.85564614	9.902288125	—0.953358011	0.00031047	0.07711271
Pdcd8	12.75065713	12.03492925	—0.715727881	0.00031379	0.07711271
Ica1	12.17364623	11.6622667	—0.511379533	0.000314358	0.07711271
LOC680426	11.2010015	10.36982724	—0.831174259	0.000315713	0.07711271

DV719080	10.87559452	9.495677787	—1.379916733	0.000318635	0.07711271
TC545343	7.438383629	6.879531146	—0.558852483	0.000332478	0.07711271
Gsta3	12.17020876	10.20865792	—1.961550836	0.00033429	0.07711271
Mfn2	10.55727808	10.01586245	—0.541415627	0.000342094	0.07711271
AABR03068037	7.785306555	7.319303528	—0.466003028	0.000351693	0.07711271
BE113961	10.80779587	9.878175532	—0.92962034	0.00035311	0.07711271
TC519727	11.59791019	10.81753792	—0.780372272	0.000357927	0.07711271
TC528585	7.453045217	6.948322568	—0.504722649	0.000359377	0.07711271
RGD1311345	8.400406852	7.683659105	—0.716747748	0.000361038	0.07711271
TC543044	11.15897338	10.80731226	—0.351661122	0.00036687	0.07711271
TC538360	10.10529859	9.492938508	—0.612360085	0.000369532	0.07711271
RGD1306053	14.18807422	12.78633165	—1.401742574	0.00037825	0.07711271
Tmem55a	10.29421099	9.628109886	—0.666101101	0.000385931	0.07711271
CK478004	4.998539607	4.108978333	—0.889561275	0.000389178	0.07711271
Cog6	8.794988406	7.924249265	—0.870739141	0.000390394	0.07711271
TC544658	11.70378121	11.34008183	—0.363699382	0.000392004	0.07711271
AA957814	7.99801766	6.667451213	—1.330566447	0.000392693	0.07711271
AA851065	10.69658123	9.076743189	—1.619838043	0.000397605	0.07711271
CB545107	10.80488687	9.566124168	—1.2387627	0.000400668	0.07711271
BP503923	11.97203025	10.95266512	—1.019365134	0.000411009	0.07711271
Dnajc8	11.88375963	11.47580858	—0.407951048	0.000412706	0.07711271
Grin2b	7.819443318	6.894573198	—0.92487012	0.000417686	0.07711271
Slc16a11_predicted	10.74873215	9.354260934	—1.394471217	0.000419341	0.07711271
Birc6_predicted	7.745470898	7.220010389	—0.525460509	0.000419662	0.07711271
Mib1_predicted	8.245212972	7.371903929	—0.873309043	0.000423797	0.07711271
TC559433	11.13137332	9.782275241	—1.349098076	0.000423917	0.07711271
AW916157	11.73649748	10.69087852	—1.04561896	0.000426872	0.07711271
AW917472	11.34281752	10.58589712	—0.756920404	0.000435355	0.07711271
Tap1	9.061885637	8.182214145	—0.879671492	0.000440342	0.07711271
AW916613	2.654368574	1.390282852	—1.264085722	0.000444876	0.07711271
Mfsd1_predicted	6.848503926	5.756632868	—1.091871059	0.000445474	0.07711271
AA818474	4.391921753	3.594459458	—0.797462295	0.000446082	0.07711271
TC563567	8.989583387	8.005430887	—0.9841525	0.000455838	0.07711271
RGD1306356	10.99949907	10.38002044	—0.61947863	0.000463089	0.07711271
DV715341	8.050123212	7.619079779	—0.431043434	0.000473159	0.07711271
BF563211	6.051837901	5.373536893	—0.678301008	0.000476289	0.07711271
Rims1	7.088218226	6.595183902	—0.493034323	0.000480843	0.07711271
AI102821	12.8265747	11.10486978	—1.721704924	0.000496254	0.07711271
Umps	10.19219914	9.598751948	—0.593447192	0.000499689	0.07711271
AI175475	6.709628141	5.644117818	—1.065510323	0.00050039	0.07711271
Nek6	8.871652763	7.678760217	—1.192892546	0.000500809	0.07711271
LOC290577	8.796783148	8.098975545	—0.697807603	0.000512747	0.07711271
Epb4.1l1	12.87470645	11.8197883	—1.054918153	0.000513513	0.07711271
AW917204	10.32038964	8.851718495	—1.468671143	0.000518431	0.07711271
Aggf1	8.624603215	7.155915536	—1.468687679	0.00052289	0.07711271
TC524569	8.967339631	8.112362678	—0.854976953	0.000530873	0.07711271
Acsbg1	14.91888151	14.34065365	—0.578227868	0.000532623	0.07711271
TC532756	7.481185857	6.76026886	—0.720916997	0.000537663	0.07711271
AW918535	14.8762029	14.0293993	—0.846803594	0.000543389	0.07711271
TC547881	8.611169112	7.435283572	—1.17588554	0.000544447	0.07711271
TC525657	7.989864402	6.913160036	—1.076704366	0.000558529	0.077922785
Dlst	14.58436665	14.05417512	—0.530191529	0.000568429	0.077922785
BE117446	4.271387612	3.642009885	—0.629377726	0.000571062	0.077922785
TC556147	11.20330191	9.935224997	—1.268076913	0.000571171	0.077922785
Rbm18_predicted	8.265474859	7.532437984	—0.733036875	0.000573185	0.077922785
Chgb	16.35659504	15.63431894	—0.722276099	0.000576277	0.077922785
LOC682205	8.396143751	7.517812838	—0.878330913	0.000576944	0.077922785
Gpr173	6.652605716	5.860519811	—0.792085905	0.000590354	0.077922785
XM_216096	7.832298665	6.566407732	—1.265890933	0.000599328	0.077922785
Ppp6c	9.764572047	9.414556134	—0.350015913	0.000605339	0.077922785
XM_343871	10.90379578	10.24265728	—0.661138501	0.000606072	0.077922785
Fgd4	8.357984576	7.228528075	—1.129456501	0.000610696	0.077922785

CX569250	9.795475601	9.227937407	—0.567538194	0.00061442	0.077922785
Hap1	12.02268819	11.01938568	—1.003302507	0.000616681	0.077922785
AW915320	9.170075196	8.417656657	—0.752418539	0.000618451	0.077922785
Rragd_predicted	10.70444865	9.093806459	—1.610642189	0.000619331	0.077922785
TC541972	10.34640635	9.316275627	—1.030130724	0.00063095	0.07819106
AI230360	7.918189901	7.234716989	—0.683472912	0.000642562	0.07819106
Igf2r	13.17561599	12.47214529	—0.703470695	0.000648788	0.07819106
DV719617	10.27750625	9.107724317	—1.169781934	0.000651766	0.07819106
Mrpl18_predicted	12.51441646	11.58262484	—0.931791612	0.000662315	0.07819106
TC568517	7.18069051	6.439772542	—0.740917968	0.000674076	0.07819106
Adsl_predicted	12.53997034	11.84135281	—0.698617527	0.000679503	0.07819106
TC519890	15.20193502	13.84576879	—1.356166231	0.000682539	0.07819106
Spg20	8.308415518	7.58305852	—0.725356998	0.000686525	0.07819106
AI170696	6.942817153	6.027001338	—0.915815815	0.00069311	0.07819106
Ube2v2	9.661199706	8.572804044	—1.088395662	0.000693451	0.07819106
LOC498295	12.20451881	11.83959221	—0.364926599	0.000698494	0.078249597
TC560921	5.207732953	4.297097796	—0.910635157	0.000700069	0.078249597
BF555924	9.985597821	9.469135844	—0.516461977	0.000702983	0.078249597
Pfkm	14.51654843	14.05002374	—0.466524695	0.000708505	0.078249597
TC564512	8.333396639	7.32224411	—1.011152528	0.000721932	0.07899534
LOC500295	9.420651641	8.540467639	—0.880184003	0.000724825	0.07899534
Sf3b2_predicted	12.44354911	11.6085621	—0.83498701	0.000725793	0.07899534
BF523428	13.15045896	12.09237333	—1.058085628	0.000726436	0.07899534
Zfp297b	8.14767076	7.796674244	—0.350996515	0.000734886	0.07936277
AA998677	6.022499947	5.66326418	—0.359235767	0.000738839	0.07936277
Commd10	13.07110695	12.22251811	—0.848588842	0.000741977	0.07936277
Usp13_predicted	8.880457276	8.301686706	—0.57877057	0.000751682	0.07936277
TC539690	11.53252368	10.19001348	—1.342510194	0.000754935	0.07936277
AW142620	10.54231861	9.200592376	—1.341726234	0.000763691	0.079628835
Keap1	12.00883633	11.60287219	—0.405964134	0.000792061	0.081218196
Gsk3b	10.9550178	10.01148948	—0.943528324	0.000797188	0.081294469
RGD1310192_predicted	10.30348412	9.743106299	—0.560377818	0.000800586	0.081294469
AW919892	9.009219476	8.398195424	—0.611024052	0.00080164	0.081294469
CX570570	9.009092379	8.150761251	—0.858331128	0.00081345	0.081955526
CO403204	7.341185431	6.396430715	—0.944754716	0.000815344	0.081955526
DV727788	9.546149034	8.506229032	—1.039920002	0.000820046	0.082157822
Crebl2	8.237847284	7.059695143	—1.178152141	0.000832899	0.082830708
RGD1566078_predicted	15.4991444	14.43680942	—1.062334982	0.000843282	0.082831536
TC536252	6.791496764	6.081404474	—0.71009229	0.000848504	0.082831536
RGD1310383_predicted	9.675442727	8.875211356	—0.800231371	0.000854032	0.082831536
Ndrg2	16.92933608	16.23958962	—0.689746461	0.000855608	0.082831536
AA819653	6.401981606	5.54072971	—0.861251897	0.000864825	0.082831536
Nr1d2	4.102851341	2.907566815	—1.195284526	0.000870343	0.082831536
AI030552	6.242106917	4.813012178	—1.429094739	0.000872853	0.082831536
TC557120	11.58985733	11.08039255	—0.509464782	0.000878637	0.083021035
CB547657	7.11769739	5.41137132	—1.706326069	0.000885415	0.083021035
TC551191	7.699104736	6.244599038	—1.454505698	0.000887765	0.083021035
Aytl2_predicted	7.999183956	7.427312229	—0.571871727	0.000891151	0.083021035
Ddef2_predicted	7.639303041	7.13151159	—0.507791451	0.000898719	0.083047388
Ratsg2	11.68645688	11.30312412	—0.383332762	0.000913048	0.083047388
ENSRNOT00000043649	12.10622909	11.2676376	—0.838591496	0.00093425	0.083047388
Slc4a4	10.52371038	9.660948829	—0.862761553	0.000935657	0.083047388
Copb2	12.96384037	12.52074638	—0.443093992	0.000945107	0.083047388
Plekhc1	9.749091344	9.407128148	—0.341963196	0.000946928	0.083047388
RGD1561030_predicted	5.157115548	4.180877924	—0.976237624	0.000950336	0.083047388
TC525601	13.50942052	12.9991972	—0.510223326	0.000950464	0.083047388
Phf7	8.191447181	7.356775616	—0.834671565	0.000953909	0.083047388
BQ782721	4.908138931	4.310952068	—0.597186864	0.000963823	0.083047388
AA925274	5.579121622	5.090008894	—0.489112728	0.000969917	0.083047388
DV727304	11.16319859	10.59010722	—0.573091369	0.000975685	0.083047388

**Table 4.** List of DEGs after H2A.Z.1 and H2A.Z.2 KD-part C (upregulated after H2A.Z.2 KD)

Symbol	Scrambled expression	shH2AZ.2 expression	log2 fold change	*p* value	FDR
Ahcyl1_predicted	11.30812916	15.70257541	4.394446257	1.55E—09	4.75E—05
Mcm6	10.83536001	11.40618674	0.570826728	8.24E—06	0.035135455
Dut	11.35492304	11.9535574	0.598634355	3.46E—05	0.066139598
Timp2	15.05728576	15.80164866	0.744362903	3.81E—05	0.067378588
RGD1311752_predicted	8.703882692	10.33537822	1.631495533	4.06E—05	0.067378588
Zcwpw1_predicted	8.549635796	9.169491306	0.619855511	4.50E—05	0.067378588
XM_345006	6.85771805	7.739541418	0.881823368	4.87E—05	0.067378588
TC549858	11.27224616	11.75359808	0.481351913	4.91E—05	0.067378588
Hrasls_predicted	1.352310448	2.473461424	1.121150976	5.02E—05	0.067378588
BF289520	5.430397248	6.059021678	0.62862443	7.69E—05	0.071367889
Pold3	10.06736193	10.48690247	0.419540544	7.71E—05	0.071367889
RGD1307357_predicted	11.2118082	11.5384726	0.326664395	7.97E—05	0.07161003
Gtf3c5_predicted	7.76009444	8.355130115	0.595035675	9.19E—05	0.073611113
Bcl7c_predicted	11.52854784	12.20876021	0.68021237	0.000109679	0.073611113
Pomc	8.615004399	9.436951714	0.821947315	0.000109978	0.073611113
RGD1565291_predicted	12.39574606	12.90176243	0.506016372	0.000117613	0.074873201
BF542749	10.74023303	12.36932877	1.629095739	0.000127234	0.077757776
Abcc2	8.819186435	9.789025783	0.969839348	0.000139329	0.080329614
LOC363188	5.75509275	8.424133782	2.669041033	0.000147266	0.083333563
Cecr5_predicted	10.95802065	11.33987144	0.381850792	0.000176782	0.08855644
AA955618	9.344852039	9.669319187	0.324467148	0.000209302	0.09181501
AI044097	5.588998691	6.267474587	0.678475896	0.000221456	0.092025775
RGD1563319_predicted	8.12622631	9.457387834	1.331161525	0.000236732	0.092741286
Tbc1d2b	6.869829194	8.039756988	1.169927794	0.000255978	0.095023281
ENSRNOT00000027501	9.948158576	10.60865914	0.660500565	0.000307587	0.095557268
RGD1304587	12.28837897	12.94341986	0.655040885	0.000314183	0.095557268
TC542265	9.893597595	10.95071148	1.05711388	0.000351139	0.095557268
BM384088	9.56848326	10.08580713	0.517323868	0.000375917	0.095557268
BG668164	9.868977302	10.4107215	0.541744198	0.000393414	0.095557268
Centg3_predicted	12.84619462	13.64912229	0.802927674	0.000397418	0.095557268
Rfc2	10.69628045	11.25875805	0.562477599	0.000401566	0.095557268
Slc26a7_predicted	4.035522477	6.194273087	2.15875061	0.000404596	0.095557268
Fn1	8.587931475	9.897009695	1.30907822	0.00041856	0.095557268
Fxyd6	15.52204053	16.22851939	0.706478863	0.000424885	0.095557268
Ezh1_predicted	13.35214855	13.9436327	0.59148415	0.000436061	0.095557268
DV719732	9.123092282	9.737560979	0.614468697	0.000443541	0.095557268
B4galt7	12.13569399	12.61112377	0.475429774	0.000458424	0.095557268
Lzts2	10.13285103	10.82559939	0.692748367	0.000469953	0.095557268
Src	9.695578825	10.36460234	0.669023515	0.000512904	0.095557268
BF556192	12.80155585	13.64456502	0.84300917	0.000526513	0.095557268
RGD1564308_predicted	8.53728578	9.316997749	0.77971197	0.00053388	0.095557268
AW915160	11.81695687	12.84151282	1.024555952	0.000534083	0.095557268
BF289433	4.810359467	5.442835512	0.632476045	0.000539006	0.095557268
LOC312502	9.813073542	10.28343955	0.470366013	0.000543607	0.095557268
Slc35b4_predicted	10.07187502	10.57533921	0.503464186	0.000547104	0.095557268
Tcn2	8.702856424	9.597660836	0.894804412	0.000548566	0.095557268
Rabl4_predicted	12.99354632	13.58209364	0.588547326	0.000558303	0.095557268
Astn1	7.130219184	7.783368618	0.653149434	0.000564477	0.095557268
Slitrk1_predicted	11.38978024	11.86215613	0.47237589	0.000565343	0.095557268
LOC678926	1.529149853	3.301916023	1.77276617	0.000568879	0.095557268
Sfmbt1	10.57247665	11.52524221	0.952765564	0.000576444	0.095557268
Nfya	7.339752471	8.230520214	0.890767743	0.000594435	0.095557268
CA503664	8.706063302	9.30753056	0.601467258	0.000612386	0.095557268
CO398332	2.088031423	3.465147675	1.377116252	0.000617647	0.095557268
Ankrd5_predicted	0.986066003	1.893556424	0.907490421	0.000618681	0.095557268
RGD1561903_predicted	8.073236386	9.261517734	1.188281348	0.000624827	0.095557268
Nav1_predicted	9.311359188	10.05118484	0.739825648	0.000627604	0.095557268
Lonrf1_predicted	6.665069586	8.231081607	1.566012021	0.000637379	0.095557268
LOC291840	10.46425437	10.98618406	0.521929682	0.00064038	0.095557268
BF555594	7.297901913	7.803911007	0.506009094	0.000642572	0.095557268
Prcp_predicted	6.945396355	7.628166927	0.682770572	0.000650274	0.095557268
Cggbp1_predicted	10.10446551	10.67233084	0.567865332	0.000651252	0.095557268

LOC686668	14.22879788	14.64664596	0.417848086	0.000653103	0.095557268
Shank1	10.5815021	11.85728804	1.275785942	0.000653256	0.095557268
BI293610	9.493734401	9.933127533	0.439393131	0.000667597	0.095557268
RGD1308637	10.63111621	10.99277702	0.361660808	0.000669694	0.095557268
LOC683646	14.6252591	15.00216313	0.376904029	0.000672344	0.095557268
TC562439	6.699930546	8.729363905	2.029433359	0.00068664	0.095625151
RGD1566063_predicted	10.42408783	10.82360645	0.399518627	0.000691139	0.095625151
XM_214416	9.178834112	9.6187083	0.439874188	0.000699314	0.095625151
RGD1308557_predicted	7.420320921	8.285465612	0.865144691	0.000733081	0.09725562
RGD1566292_predicted	11.21332683	11.71063736	0.49731053	0.000751031	0.097309775
RGD1305166_predicted	9.134297704	9.759187045	0.624889341	0.000751656	0.097309775
Nkiras2_predicted	10.80772401	11.77210764	0.964383627	0.000753238	0.097309775
Cybasc3	7.043310663	7.609593274	0.566282611	0.000754734	0.097309775
Mrpl36_predicted	11.89399742	12.33502107	0.441023645	0.000782062	0.098010329
Gprk2l	8.779110352	9.38417973	0.605069378	0.00080672	0.098999718
LOC686766	5.260876485	5.911787655	0.65091117	0.000811953	0.099243336
Phf22	7.538016027	8.324087108	0.786071082	0.000836521	0.101357181
RGD1306143_predicted	6.850673885	7.335015831	0.484341946	0.00084597	0.101533717
ENSRNOT00000048826	12.49598977	12.94226435	0.446274573	0.000847305	0.101533717
Znf579_predicted	11.20811184	11.67550922	0.467397383	0.000856332	0.10181686
Hsdl1	9.808903868	10.14043298	0.331529114	0.000883112	0.102944694
Gprk6	12.47356751	13.25494457	0.781377066	0.0008894	0.102944694
Hsd17b2	3.414854355	5.901570573	2.486716218	0.000943101	0.104035619
Rnf8	12.29803729	12.87630368	0.578266392	0.000952234	0.104035619
CA510534	11.34797575	12.80389432	1.455918574	0.000958972	0.104035619
Arl2	10.91134235	11.45372552	0.542383164	0.000971266	0.104035619
BF550209	12.37525589	12.8730042	0.497748308	0.000974014	0.104035619
BG665133	11.20679244	12.33422623	1.127433798	0.000979781	0.104035619
LOC691075	9.060232543	9.478672438	0.418439896	0.000980214	0.104035619
RGD1308535_predicted	9.816766494	10.28292832	0.46616183	0.000993396	0.104035619
Zswim5_predicted	5.470774971	6.301634314	0.830859343	0.000996038	0.104035619
LOC690354	10.40224677	10.7558671	0.353620329	0.000997294	0.104035619

**Table 5.** List of DEGs after H2A.Z.1 and H2A.Z.2 KD-part D (downregulated after H2A.Z.2 KD)

Symbol	Scrambled expression	shH2AZ.2 expression	log2 fold change	*p* value	FDR
Rnf14	16.57094576	12.9463825	—3.624563259	1.91E—08	0.000291288
RGD1359108	13.30079953	7.879686571	—5.421112956	4.85E—08	0.000494164
Inpp5f_predicted	4.824671522	3.588112893	—1.236558629	1.13E—06	0.008652283
Cfl2_predicted	10.28475755	8.941614741	—1.343142814	5.78E—06	0.033552658
Tex2	6.820637729	5.774872776	—1.045764953	6.59E—06	0.033552658
Lypla3	10.13489074	9.305455152	—0.829435583	9.20E—06	0.035135455
Sbds	10.29127274	9.876346931	—0.414925805	1.21E—05	0.041035544
Poldip2_predicted	11.21978549	10.11515746	—1.104628032	1.36E—05	0.041433838
RGD1564626_predicted	7.402821848	6.749656546	—0.653165302	1.94E—05	0.05398694
Chchd3_predicted	13.15554854	11.50732751	—1.648221032	2.44E—05	0.062079366
Wee1	7.228033394	5.956374529	—1.271658865	2.65E—05	0.06223942
U78138	8.370979606	6.934655598	—1.436324008	3.15E—05	0.066139598
XM_343572	12.87985279	12.26365661	—0.616196176	3.39E—05	0.066139598
AI231051	5.91510764	4.336231214	—1.578876425	5.07E—05	0.067378588
Gad2	7.493926937	5.159298815	—2.334628122	5.51E—05	0.06840792
Tmem55a	10.29421099	9.510629962	—0.783581025	5.80E—05	0.06840792
Lsm14a_predicted	8.399662619	7.415627465	—0.984035154	6.05E—05	0.068497749
LOC300429	6.310030902	5.753092672	—0.556938229	6.85E—05	0.071367889
Sdcbp2	3.853370711	3.05190314	—0.801467571	6.94E—05	0.071367889
LOC691853	9.783324367	8.799255704	—0.984068662	7.16E—05	0.071367889
XM_215270	10.17499295	9.701596351	—0.4733966	7.43E—05	0.071367889
TC538215	9.067975732	7.252392924	—1.815582808	8.86E—05	0.073611113
BG663025	6.859758488	5.74663244	—1.113126048	8.89E—05	0.073611113

TC553657	8.723092234	8.029121219	—0.693971015	9.42E—05	0.073611113
LOC684322	15.44820244	14.34666049	—1.101541943	9.84E—05	0.073611113
Usp32_predicted	10.25821098	9.517372161	—0.74083882	0.000100946	0.073611113
ENSRNOT00000008889	8.21062512	6.810763402	—1.399861718	0.000104197	0.073611113
Rb1	8.11762273	6.908923008	—1.208699722	0.000105314	0.073611113
AA849985	6.116135406	5.241886237	—0.874249169	0.000110254	0.073611113
AW917590	14.02219537	12.7498481	—1.272347268	0.000111886	0.073611113
CB547899	7.72064826	7.029262406	—0.691385855	0.000113222	0.073611113
U78132	7.279222721	5.846742231	—1.43248049	0.000121025	0.075472833
Mcf2l	9.397430132	8.657675863	—0.739754268	0.00013013	0.077968283
DV713600	13.15808683	11.11117444	—2.046912388	0.00013701	0.080329614
TC539285	9.30257925	8.414415448	—0.888163802	0.000151429	0.084131469
BF555161	10.59364955	9.409913061	—1.183736486	0.000167666	0.087621405
Cdk5rap1	10.22213249	9.608946578	—0.61318591	0.000168425	0.087621405
CO399145	7.567286017	6.640966202	—0.926319815	0.000169575	0.087621405
BF544403	7.874729806	6.432614592	—1.442115214	0.000172048	0.087621405
RGD1308147	9.865899439	8.842423951	—1.023475489	0.000183836	0.090604523
Arl10	7.253170158	6.41752538	—0.835644778	0.000195782	0.09181501
Ddn	11.23443915	9.493619755	—1.740819393	0.00019651	0.09181501
AW535924	2.716704964	1.208805749	—1.507899216	0.000200473	0.09181501
AW917511	7.921316801	7.098393914	—0.822922887	0.000207115	0.09181501
Atxn1	7.381331792	6.174416218	—1.206915574	0.000210514	0.09181501
Eaf2	5.106563436	4.649076352	—0.457487084	0.000211747	0.09181501
TC557676	7.472657949	6.19779289	—1.27486506	0.000212226	0.09181501
Ttc12	7.900994459	7.523648839	—0.37734562	0.000213335	0.09181501
TC560921	5.207732953	4.559434897	—0.648298056	0.000217244	0.092025775
AI112975	6.352713186	5.466730174	—0.885983012	0.000228285	0.092025775
Vil2	9.860779156	8.967669681	—0.893109474	0.000228882	0.092025775
AA875008	5.494818563	4.700887004	—0.793931559	0.000235511	0.092741286
TC528454	8.35589654	6.885831687	—1.470064854	0.000247959	0.09493033
Hspca	14.42409811	13.70998635	—0.714111765	0.00025058	0.09493033
AI059618	4.100124355	1.829444923	—2.270679431	0.00025164	0.09493033
Rnf11_predicted	10.2186121	9.615662304	—0.602949799	0.000258106	0.095023281
Pole3	12.55132532	11.31822759	—1.233097729	0.000262232	0.095273647
RGD1564451_predicted	9.713981828	8.589317234	—1.124664593	0.000265021	0.095273647
LOC683512	10.77887127	9.314774611	—1.464096657	0.000269989	0.095557268
Stx6	12.20386283	11.83254524	—0.371317584	0.000275093	0.095557268
TC539851	11.07018722	9.38957993	—1.680607294	0.0002773	0.095557268
RGD1308581_predicted	11.33094265	10.731295	—0.599647641	0.000301272	0.095557268
RGD1305052_predicted	12.11367655	10.94947524	—1.164201304	0.000303392	0.095557268
Pde4a	9.320418187	8.426565098	—0.893853089	0.000307772	0.095557268
RGD1565785_predicted	3.454975278	1.716774529	—1.738200749	0.000308741	0.095557268
TC551503	10.21507032	9.012702885	—1.202367432	0.0003089	0.095557268
Sdhd	14.37797406	13.64470284	—0.733271224	0.00031574	0.095557268
AW144312	10.73124208	8.983532052	—1.747710029	0.000316237	0.095557268
AW144351	9.197071628	7.899938415	—1.297133212	0.000317995	0.095557268
AI599930	7.678810398	7.029318808	—0.64949159	0.0003243	0.095557268
Ssu72	10.81988308	10.15157164	—0.66831144	0.000326611	0.095557268
MGC125215	13.54599663	12.92503148	—0.620965157	0.000330105	0.095557268
TC524569	8.967339631	8.237038714	—0.730300917	0.000331746	0.095557268
Inpp4a	8.581584747	7.638446802	—0.943137945	0.000332111	0.095557268
TC540740	12.032179	11.48000894	—0.552170063	0.000338226	0.095557268
TC550497	12.02106544	11.01957227	—1.001493172	0.00034439	0.095557268
XM_580045	8.850520618	7.850512112	—1.000008505	0.0003564	0.095557268
XM_236784	11.16551643	10.17544134	—0.990075092	0.000365511	0.095557268
AW915589	9.355873567	7.887143897	—1.46872967	0.000371323	0.095557268
Garnl1	6.899821493	6.075097502	—0.824723991	0.00038265	0.095557268
H33079	11.05742197	10.63726213	—0.420159837	0.000383244	0.095557268
LOC689577	10.30988195	9.693679501	—0.616202449	0.000385184	0.095557268
LOC683630	9.849902817	9.088044225	—0.761858591	0.000385627	0.095557268
Arpc5	14.5853404	13.66460753	—0.920732865	0.000387329	0.095557268
RGD1305797_predicted	5.300372467	4.356613034	—0.943759433	0.000389433	0.095557268
TC546991	7.224586672	5.935472523	—1.289114149	0.000393929	0.095557268

TC538360	10.10529859	8.83610561	—1.269192983	0.000408086	0.095557268
Gdi2	11.96894942	10.24844069	—1.720508726	0.00041582	0.095557268
BM391850	6.763729833	6.106887394	—0.656842439	0.000416848	0.095557268
Habp4_predicted	12.75635898	12.10459874	—0.651760235	0.000419683	0.095557268
TC541875	10.06553783	9.450612436	—0.614925399	0.000440675	0.095557268
DV718172	13.52733676	12.71122856	—0.816108202	0.000445019	0.095557268
Pgam5	8.963640697	8.159415155	—0.804225543	0.0004591	0.095557268
TC557253	11.21445231	9.296227403	—1.918224906	0.000461312	0.095557268
Gs3	9.481151768	8.642831162	—0.838320607	0.000463394	0.095557268
LOC681501	11.05409438	10.18583791	—0.868256472	0.000473823	0.095557268
BF281819	13.04857617	11.8092362	—1.239339965	0.000477166	0.095557268
CX569261	12.28234494	10.68729318	—1.595051767	0.000484223	0.095557268
CK596627	11.19585087	10.35777472	—0.838076159	0.000485026	0.095557268
Eif2c1_predicted	8.545178383	8.070863133	—0.47431525	0.000487557	0.095557268
DV726726	11.92676689	11.51677995	—0.409986941	0.000496554	0.095557268
Nefh	8.341691009	6.745856633	—1.595834376	0.000498525	0.095557268
Fahd1	9.969599036	9.383759066	—0.585839969	0.000500438	0.095557268
Tacc1	9.627935629	8.305618957	—1.322316672	0.000502046	0.095557268
Ppp3r1	9.456090327	8.882689108	—0.573401219	0.000504727	0.095557268
AW920896	8.970038953	8.300730935	—0.669308018	0.000505679	0.095557268
Atp6v1h	8.289085697	7.934631332	—0.354454365	0.000506174	0.095557268
LOC310395	10.4119975	9.308354087	—1.103643409	0.000510548	0.095557268
Dcbld2	6.201411422	5.130397482	—1.07101394	0.000512876	0.095557268
Hspa9a_predicted	11.06428449	9.911168244	—1.153116244	0.000529115	0.095557268
TC563075	7.096430274	5.632571768	—1.463858506	0.000532538	0.095557268
TC562837	7.37169182	5.1955973	—2.17609452	0.000536894	0.095557268
Mkks	9.065711002	8.556237021	—0.509473981	0.000544585	0.095557268
Mapk15	5.821049535	5.019106294	—0.801943241	0.000545662	0.095557268
Snx27	9.577525457	8.670999828	—0.906525629	0.000550679	0.095557268
TC523914	5.475100961	4.273483854	—1.201617107	0.000559079	0.095557268
AI029023	12.72038418	10.95540125	—1.764982934	0.000577539	0.095557268
Abcg3	5.995608197	4.88764231	—1.107965887	0.000580682	0.095557268
TC540837	7.653046158	6.293159397	—1.359886761	0.000583829	0.095557268
CB718612	8.141380063	7.502377182	—0.639002881	0.000586497	0.095557268
CB547822	7.48574771	7.056202285	—0.429545426	0.000586655	0.095557268
Cxxc6_predicted	5.461267196	3.727894207	—1.733372989	0.000596532	0.095557268
AW917678	8.008522785	7.106552387	—0.901970397	0.000598751	0.095557268
Dlgap2	6.739059513	6.048951702	—0.690107811	0.000612053	0.095557268
RGD1305269_predicted	10.48363487	9.989398981	—0.494235885	0.000618636	0.095557268
TC543111	5.988905601	5.175476656	—0.813428946	0.000624841	0.095557268
AW915587	12.81417625	12.0617611	—0.752415147	0.000629005	0.095557268
LOC302495	8.838373315	6.8520573	—1.986316015	0.000632419	0.095557268
Arhgef9	10.96945893	10.06784789	—0.901611047	0.000635486	0.095557268
Atp5b	15.39132483	15.05979828	—0.331526546	0.000641824	0.095557268
AI044115	4.38279618	3.418203792	—0.964592388	0.000651088	0.095557268
TC523083	6.820128764	6.144807662	—0.675321103	0.000654296	0.095557268
Rab11fip4_predicted	8.321833638	7.268975447	—1.052858191	0.000661426	0.095557268
TC523910	7.889138456	6.19649015	—1.692648306	0.000661665	0.095557268
BE108224	3.824896985	1.37170849	—2.453188495	0.000669059	0.095557268
LOC499900	9.327866735	8.709208418	—0.618658317	0.000670927	0.095557268
TC537999	6.001132395	4.678122045	—1.32301035	0.000676124	0.095625151
RGD1308745_predicted	4.568246264	3.179251688	—1.388994575	0.000687651	0.095625151
LOC688553	3.191286936	1.693084684	—1.498202253	0.000696309	0.095625151
Morn1	7.972102174	6.856300055	—1.115802119	0.000699611	0.095625151
TC530505	4.769710018	3.940413803	—0.829296214	0.000710803	0.096012504
BF396589	4.318891305	3.588142974	—0.730748331	0.000713252	0.096012504
CO554673	9.464355537	8.372142043	—1.092213494	0.000729299	0.09725562
TC543488	5.436948213	2.78354383	—2.653404383	0.000730873	0.09725562
TC532226	6.929504114	4.480025699	—2.449478415	0.000735218	0.09725562
TC566921	8.943896089	7.990664073	—0.953232016	0.000747355	0.097309775
AW914885	10.02209083	9.545298515	—0.476792314	0.000763893	0.097903454
BF556147	7.287577236	6.440436939	—0.847140297	0.000765747	0.097903454

Trim2	12.92583443	12.5169377	—0.408896732	0.000769469	0.097969457
Gpr158_predicted	6.695389757	5.535821733	—1.159568025	0.000782843	0.098010329
BG666117	12.11075957	11.62393319	—0.486826385	0.000784673	0.098010329
TC541992	7.227521264	6.312830078	—0.914691186	0.000785637	0.098010329
TC525657	7.989864402	7.075628128	—0.914236274	0.000787993	0.098010329
TC560235	8.131101996	7.601540913	—0.529561083	0.000804594	0.098999718
LOC685076	11.93684157	10.40645356	—1.53038801	0.000818584	0.099655287
AW143088	11.05925856	10.17888615	—0.88037241	0.000852562	0.101764579
TC537691	11.03421255	10.1832052	—0.851007351	0.000872934	0.102944694
Clcn2	10.18273395	9.654510577	—0.528223376	0.000877455	0.102944694
DV719080	10.87559452	9.843234019	—1.032360501	0.000877857	0.102944694
DY471780	7.546202473	6.688303944	—0.857898528	0.000885155	0.102944694
Ppm1e	8.969105775	7.850748852	—1.118356922	0.000904556	0.103853197
TC538748	11.28808193	10.56735898	—0.720722956	0.000907445	0.103853197
AI170363	4.98690796	4.240445668	—0.746462292	0.000917269	0.104035619
TC523584	8.251731137	7.876311518	—0.375419619	0.000921387	0.104035619
AI145492	4.960957943	3.550461441	—1.410496501	0.000927701	0.104035619
CF109839	7.043330761	6.236742364	—0.806588397	0.000929916	0.104035619
TC559053	8.399715145	7.440335331	—0.959379814	0.000932371	0.104035619
AB040488	4.509816182	3.935696141	—0.574120041	0.000936815	0.104035619
CB544681	7.403628364	6.79873552	—0.604892844	0.000949976	0.104035619
Arhgef11	8.620841844	8.014495747	—0.606346097	0.00096325	0.104035619
AA800571	8.989335176	8.145442592	—0.843892585	0.000970797	0.104035619
AA965181	6.11182251	4.75033957	—1.36148294	0.000977883	0.104035619
Ripk5	6.33665685	5.821877894	—0.514778956	0.000978231	0.104035619
Phka1	5.544744433	4.356297924	—1.188446509	0.000984497	0.104035619
TC524191	12.34462962	11.67308416	—0.671545452	0.00098735	0.104035619
BG663084	9.722716915	8.835969466	—0.886747449	0.000995922	0.104035619
BP503923	11.97203025	10.96018715	—1.011843108	0.00099756	0.104035619

Collectively, our current studies indicate that H2A.Z hypervariants mediate activity-induced IEG transcription in a context-dependent (H2A.Z.1) or context-independent (H2A.Z.2) fashion, where the extent of their involvement in the transcriptional process varies from gene to gene. Our data also suggest spatial and temporal cooperation of these hypervariants in manifesting the maximum gene response under a given circumstance. Natural extensions of the current work will be to test the above hypothesis about allele/cell-specificity of these hypervariants, and to investigate molecular mechanisms connecting H2A.Z hypervariants and paused Pol II. Additionally, sequentially mutating H2A.Z.1 to H2A.Z.2 could provide insight into which amino acid differences are necessary for functional specificity. Particular attention may be paid to the H2A.Z.1^14S^/H2A.Z.2^14A^ variation, where H2A.Z.1, but not H2A.Z.2, could be potentially phosphorylated. It will also be of interest to learn about hypervariant-specific H2A.Z chaperones, if any, and understand how these hypervariants differentially regulate expression of several crucial synaptic molecules. Finally, it is our hope that the data presented here will increase appreciation for functional specificity of H2A.Z hypervariants and promote tool-building efforts to enable a comprehensive understanding of diverse roles played by these paralogs at different genes and under different neurobiological contexts.

## Synthesis

Reviewing Editor: Leonard Maler, University of Ottawa Decisions are customarily a result of the Reviewing Editor and the peer reviewers coming together and discussing their recommendations until a consensus is reached. When revisions are invited, a fact-based synthesis statement explaining their decision and outlining what is needed to prepare a revision will be listed below. The following reviewer(s) agreed to reveal their identity: Ian Maze

The reviewers both agree that this is now a well done and useful addition to the literature on the regulation of IEGs. One reviewer finds that some controls are not accurately described and some results over-interpreted. This reviewer gave many detailed suggestions on how to improve this manuscript so that it is suitable for publication in eNeuro and I have copied these comments, in full, below.

The authors should address all the points made below and carefully respond with details of their revisions. This Ms will not require re-review but I will carefully go over the revised Ms and the authors' detailed response to this review. If the revisions prove complete, I will be able to accept this Ms for publication in eNeuro.

Reviewer #2

The authors present a compelling set of data describing the gene specific targets of H2A variants. In general, I believe this dataset will be of interest to those who work on H2A.Z and IEGs and their regulation of expression. While interesting, several aspects of uncontrolled or over interpreted results slightly diminish my enthusiasm. I suggest ways to fix some of the manuscript. However, the entire document should be closely looked at.

Overview of review: 

• I am mostly concerned about the lack of controls related to ChIP experiments. In general, ChIP data is difficult to interpret and even more so without antibody controls or controls for whether the antibody precipitates the intended target. For these experiments, I think the authors should be more specific about what they did (including the exact antibody used) as opposed to making conclusions about what they think is going on. 

• There are slight inconsistencies with some data that do not seem to be addressed. For example, the H2A mediated control of Arc levels does not seem to line up with the H2A mediated control of synaptic activity (see below). The authors should specifically address these data and how they are consistent. 

• The authors should show a time line for treatments and measurements. There are many experimental approaches in this manuscript that are trying to say something similar and I found it difficult to determine whether I was looking at data from neuronal cultures, what the time frame of treatment was, type of culture etc. 

• If the authors are going to say anything about changes to an antibody signal on an immunoblot it should be accompanied by quantification unless it is truly obvious in multiple examples such as that seen in Figures 4D and 4E. 

Specifics of review: 

Figure 1B: Why does every nucleus not show H2afz or H2afv staining? Is this a technical limitation? One possibility is that this is due to cell specific expression of these variants. 

1. How many times has this been done? 

2. The authors should quantify these puncta in some manner? 

3. The authors should quantify the overlap? 

Figure 1D-G: MW markers please. Full representative westerns for each antibody should be shown in a supplemental file. 

Figure 3A: These data appear convincing. How are these experiments being blinded? Was any ICC done to confirm that the recorded cell was knockdown for the respective H2A. Not necessary but is good practice especially because it seems as though from Figure 1B that not every cell has each H2A variant. Also, it is unclear how long the cells were transfected before mEPSC recordings. This is important information as changes in gene expression can occur considerably faster than remodeling of the synapse. 

Figure 3D: The immunoblot analysis looks as though there are no changes. I am unclear as to what the authors are trying to say with this data. The Shank3 antibody does not look convincing. A knockout control would be needed to confirm this antibody. To say anything substantial about this data, quantification of several westerns would be needed. The actin signal looks overexposed and thus would be hard to normalize. Genomic analysis is many times more sensitive than protein analysis, and many many times more than immunoblot analysis. Immunoblots are not a great tool for detecting small changes. It is important that with such small changes the authors take care in quantifying such data or making appropriate interpretations from the unquantified data. It seems that the authors do not need this data and it could be taken out. It is barely mentioned/discussed in the text and does not line up well with the gene expression data. Stating that the antibody signal is modestly reduced does not mean anything. The signal is clearly unchanged. Also, I am sure the authors are aware that immunoblots do not show protein levels they show antibody signal. Determining protein levels by immunoblot requires comparison to knockout controls to determine specificity of signal followed by careful quantification of signal based on a control signal. Then one needs to use immunoblot techniques that have the capacity to detect small changes (i.e. I125 labeled antibody or fluorescent imaging technology). Alternatively, one could use SILAC quantitative mass spec to measure protein level in their different samples. 

Figure 4: These data indicate that loss of H2A variants leads to a decrease in activity induced Arc expression. One would predict that this would lead to the inability to recycle GluR1 (i.e. more surface GluR1). This should then lead to increased synaptic activity. However, the authors show that reduced H2A variants in Figure 3 have reduced postsynaptic signaling. What are the authors thought on this? 

Figure 6: Is there equal pulldown of Myc or HA in treated ChIP samples as compared to untreated. This data could arrive from just having less protein coming down in the precipitated samples. Not sure I understand this data correctly in context of other findings. If Bic+AP leads to a reduction in H2A binding to Arc (Figure 6C), how is H2A required for activity induced Arc expression (figure 4D)? 

Figure 7: Where are the controls showing that these antibodies can immunoprecipitate the intended proteins? This data is not interpretable without such information. Alternatively, the authors could do the ChIP in the knockout for each protein to confirm that the information being measured is indeed specific to the protein being pulldown. Otherwise, I do not believe anything can be learned from these data. Moreover, I object to the over interpretation in this section. Fore example, the authors state the following on lines 543-547: 

“Here, we tested whether there is a relation between Pol II pausing and H2A.Z hypervariants at the Arc TSS. Using ChIP with anti-RPB1 (N-terminus), the largest Pol II subunit, we found that RNA Pol II pausing at the Arc TSS - notable from the enrichment of RPB1 near nucleosome +1 at the TSS (primer 2 in Figure 7D) - is diminished significantly in neurons depleted of H2A.Z.2, but not H2A.Z.1.” 

Besides being a complicated way to state their point there are several issues with the interpretation that RNA Pol II pausing at the Arc TSS is happening based on the data. Experimentally, the authors applied antibody raised against the N-terminus of RPB1 to their neuronal samples prepared as described in their ChIP protocol. They then immunoprecipitated these samples and detected Arc signal by qPCR. Based on the data, they detected less Arc signal in anti-RPB1 immunoprecipitates derived from of ChIP lysates that were previously transfected with H2A.Z.2 shRNA, but not H2A.Z.1. Here are some possible ways in which this could have happened: 

1. The antibody to RPB1 has diminished capacity to precipitate RPB1 protein in samples previously transfected with H2A.Z.2 thus you get less Arc signal. 

2. The antibody to RPB1 precipitates a complex of proteins of which another protein interacts with the Arc TSS and is no longer does so when H2A.Z.2 shRNA has been transfected. 

3. The H2A.Z.1 shRNA did not knockdown this variant in these experiments. 

4. This is a completely non-specific immunoprecipitation that artificially interacts with Arc DNA and is affected by H2A.Z.2. 

I like figures 8, 9, 10 and 11
